# How do non-radiologist physicians read and use the radiology report? A French national survey

**DOI:** 10.1186/s13244-026-02387-1

**Published:** 2026-09-05

**Authors:** Yasmine Kassab, Matthieu Bailly, Sixtine Brabant, Catherine Oppenheim, Alice Bernard-Tessier, Natacha Stolowy, Helene Salaün, Maelle Dejobert, Thibaut Lemoine, Marine Begon, Grégoire Boulouis, Jean-Philippe Cottier, Marie-Agnes Lauvin, Helena Daurel, Anne-Laure Piton, Raoul Kanav Khanna, Gilles Metrard, Clara Cohen

**Affiliations:** 1 Department of Neuroradiology, University Hospital of Orléans, Orléans, France; 2 Department of Nuclear Medicine, University Hospital of Orléans, Orléans, France; 3 Laboratoire Interdisciplinaire pour l’Innovation et la Recherche en Santé d’Orléans, University of Orléans, Orléans, France; 4 République Française Ministère du Travail de la Santé des Solidarités et des Familles, Paris, France; 5 IMA-Brain, Institute of Psychiatry and Neuroscience of Paris (IPNP), INSERM U1266, Université Paris Cité, Paris, France; 6 Department of Neuroradiology, Hôpital Sainte-Anne, GHU-Paris Psychiatrie et Neurosciences, Paris, France; 7 Department of Medical Oncology, Gustave Roussy Institute, Villejuif, France; 8 Department of Ophthalmology, Assistance Publique Hôpitaux de Marseille, University Hospital La Conception, Marseille, France; 9 CEMEREM Laboratory, UMR 7339, Aix-Marseille University, Marseille, France; 10 Department of Medical Oncology, Institut Curie, PSL Research University, Paris, France; 11 Hôpital privé de l’Est Lyonnais, Radiology, Imagerie médicale du sud Lyonnais (IMSEL), Lyon, France; 12 Emergency Department, Regional Hospital Center of Gien, Gien, France; 13 Department of Diagnostic and Interventional Neuroradiology, Regional University Hospital Centre Tours, CIC-IT 1415, Tours, France; 14 University of Tours, INSERM, Imaging Brain & Neuropsychiatry iBraiN U1253,, Tours, France; 15 Regional University Hospital Centre Tours, Centre d’Investigation Clinique - Innovation Technologique (CIC-IT) 1415, Tours, France; 16 Neuroradiology and Adult Radiology Department, Regional University Hospital Centre Tours, Tours, France; 17 Medical Imaging Group 37, Tours, France; 18 Medical Imaging Group 45, Orleans, France; 19 Department of Internal Medicine, University Hospital of Orléans, Orléans, France; 20 Department of Ophthalmology, Regional University Hospital Center of Tours, Tours, France; 21 INSERM, Imaging Brain & Neuropsychiatry iBraiN U1253, Tours, France

**Keywords:** Radiology report (RR), Non-radiologists physicians, Preliminary reports, Key images, Medical error

## Abstract

**Objectives:**

We developed a national questionnaire to assess radiology reports (RR) physicians’ reading habits and identify their expectations towards RR, focusing on CT and MRI reports.

**Materials and methods:**

An anonymized 20-item questionnaire was nationally distributed in France via practice and hospital mailing lists and one social media group, and included three sections: respondent’s profile; RR reading (importance and reading of RR sections, advice on key images [i.e., selected summary screenshots for main findings]); use and impact of the RR. Overall and profile-based subgroup analyses were performed.

**Results:**

One thousand two hundred fifty physicians responded (58% women, 71% aged 20–34 years, 53.4% juniors, 85% from medical specialties). RR reading: 60.3% frequently read the whole report, 17.4% conclusion-only, and 9.9% images-only. Most respondents considered key images essential for comprehension, positively influencing opinion on RR and reinforcing confidence in the conclusion; 63.3% agree with a systematic key-images inclusion within RR. Concerning preliminary reports, 49.3% await validation by a senior radiologist to make their decision. RR use and impact: 55.7% copy the radiologist’s conclusion into their record, 39.5% reformulate it. Seven percent admitted having made a medical error by not reading the RR carefully. In life-threatening emergencies, 75% review the RR retrospectively, 18.5% refer directly to specialists regardless of the radiologist’s opinion. Responses were influenced by respondents’ profile (medical specialists vs surgeons, academic status, experience, gender), especially regarding the importance of the radiologist’s name and the incorporation of the conclusion.

**Conclusion:**

Most clinicians declared reading the entire RR. Key images add value, while clinician profile shapes the way they read and use RR.

**Key points:**

- **Question:** How do clinicians read and incorporate RR into medical records, and does the clinician’s profile influence these practices?
- **Findings:** Out ot 1253 respondents, 60.3% read the entire report or the findings section depending on the conclusion; 55.7% copy the radiologist’s conclusion.
- **Critical relevance statement:** RR’s conclusions are commonly incorporated as is into the record. Careful conclusion formulation, together with the addition of illustrating key images, may facilitate report use. These findings prompt consideration of tailoring reporting practices to the profile of the requesting physician.

**Graphical Abstract:**

**Figure:**
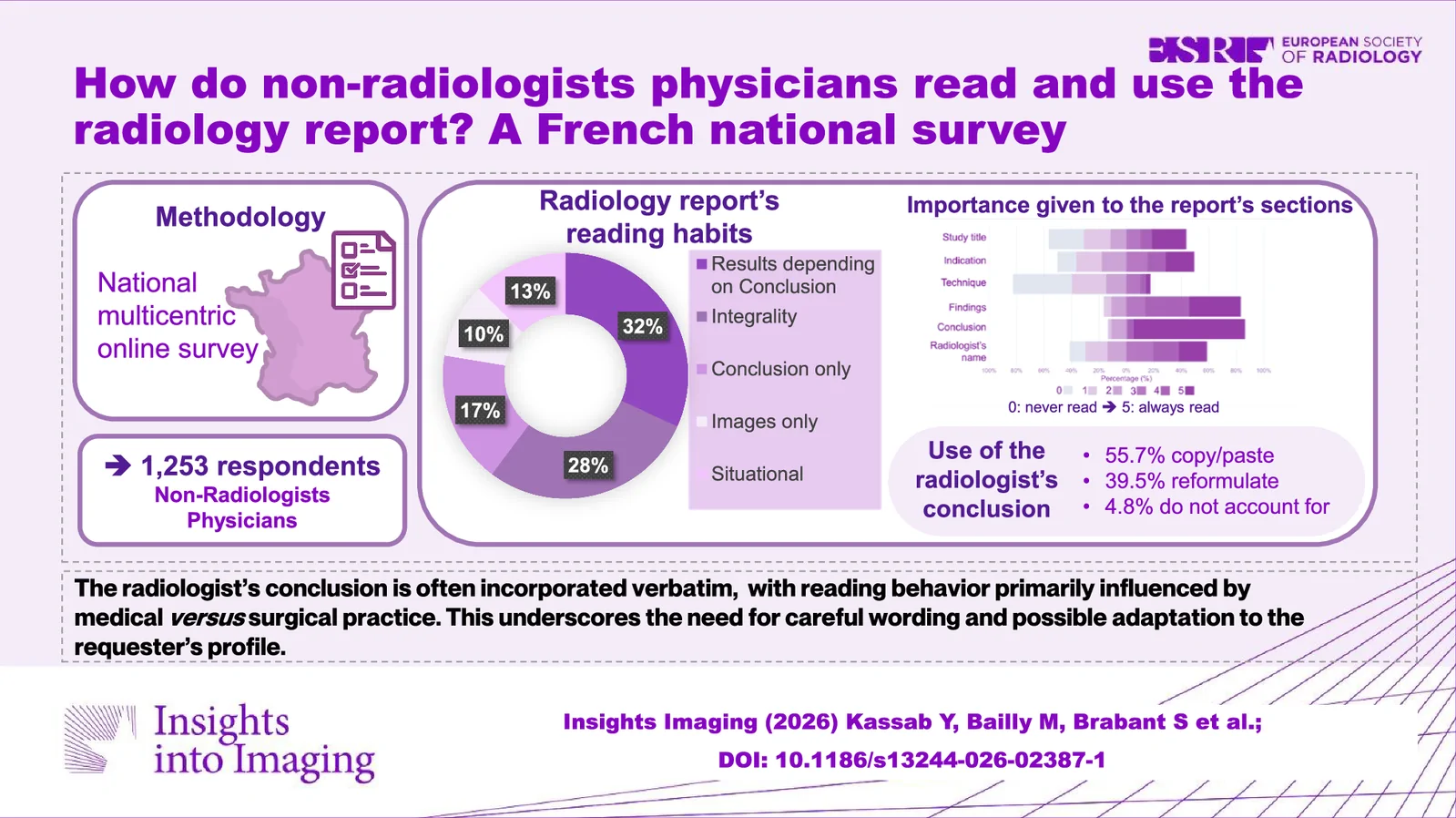

## Introduction

Radiology reports (RR) are a cornerstone of communication between radiologists and clinicians and play a crucial role in guiding clinical decision-making [1]. The role of the radiologist is therefore fundamental, particularly in crafting precise and comprehensible reports, essential for optimal care. Nevertheless, the RR drafting remains highly subjective, shaped by individual practices and experiential learning [2].

The adoption of structured and standardized RR, while promising, has faced barriers to widespread implementation [3–6]. Challenges such as rigidity in accommodating complex cases and technical issues that hinder seamless integration into clinical workflows have limited its uptake [7]. Meanwhile, the incorporation of visual elements such as key images (KI), despite their well-documented benefits for enhancing understanding and informing medical decision-making, remains partially explored in current research [8–12].

Numerous studies have investigated the RR quality criteria, emphasizing the importance of concise and clear communication [13]. However, the expectations, reading habits, and challenges faced by non-radiologist physicians vary depending on their specialties and the type of healthcare facilities they work in. Despite this, relatively few studies have focused on how clinicians (i.e., non-radiologist physicians) read and utilize these reports in practice [14*–*21]. The need to give greater consideration to referring clinician and patient preferences has been highlighted [22].

How physicians read and use RR has long been questioned but only sporadically investigated. Early work showed generally positive attitudes toward RR, with substantial variability in reading habits, some clinicians reading full reports, and others focusing mainly on conclusions [14]. Subsequent surveys confirmed this heterogeneity [23]. Studies have consistently shown that clinicians value detailed interpretations and often appreciate radiologists’ management suggestions, though many still prefer to review images themselves [18]. More recent data indicate that most physicians do read the full RR, with demographic factors such as sex and experience influencing reading behavior; for example, females are more likely to read entire reports and more experienced physicians are more inclined to review native images in addition to the text [18, 24]. Specialty-specific surveys reveal further nuances: orthopedic surgeons may forgo reports for standard radiographs but rarely for cross-sectional imaging, and many express the need for structured elements such as angle measurements of KI [20]. A survey of academically oriented clinicians showed strong support for medical imaging, but their research-focused profiles limit how well they represent broader everyday clinical practice [25].

Some previously cited studies involve relatively small sample sizes and/or limited representation of subspecialties, making it difficult to analyze behaviors specific to individual specialties or demographic groups. Additionally, the use of the RR once made available to referring physicians has not been directly explored, and the added value of KI (i.e., selected images embedded within the report) has been sparsely investigated [8, 11, 12].

Our study aims to explore how clinicians read and incorporate CT and MRI RR, with a focus on key factors such as the role of diagnostic hypotheses, the added value of KI, the importance of preliminary reports, the utilization of reports, and variations in reading practices based on the reader’s profile. By conducting a national survey of hospital and private practice clinicians, this research seeks to gain deeper insights into their expectations and practices.

## Materials and methods

### Survey design

This study, which received approval from the national imaging review board (Comité-d’Ethique-de-Recherche-en-Imagerie: CRM-2412-440), is a national multicenter prospective cross-sectional survey, developed with a public health physician (S.B.), constructed in accordance with STROBE recommendations [20, 26]. The need for approval was waived, as respondents were informed that their data would be collected and analyzed anonymously for the purpose of scientific communication and publication. The survey was administered via Microsoft Forms (Microsoft Office 365, Version 2403, 2023) and used a 20-item anonymized questionnaire. It was distributed from July 2023 to February 2024, via institutional mailing lists or social media, to 16,983 physicians. The coordinating center sent invitations in July with reminders in October; other institutions sent invitations in October and January, followed by reminders four weeks later. The survey targeted physicians from multiple specialties across 13 institutions (*n* = 1983): four university hospitals, one regional hospital, two private non-profit facilities, six clinics and one Facebook group (“French residents”, *n* = 15,000), covering several French regions.

### Questionnaire development

Our primary objective was to develop a framework describing how non-radiologist physicians read and use RR in daily practice, with the aim of gaining deeper insights into their behaviors and needs.

The questionnaire was structured around key domains: the practical reading of RR, focused on CT and MRI reports, attitudes toward KI and preliminary reports, the use of RR in clinical decision-making and medical records, and potential variations in behavior in emergency settings. As it was not feasible to directly observe how clinicians interact with RR in real-life practice, we designed a pragmatic questionnaire, seeking to formulate questions that were sufficiently specific to provide meaningful information while remaining relevant to everyday medical practice.

The questionnaire was designed to address the following main research questions:

- Are the respondents representative of the broader medical community, and does radiology report use vary according to the clinician’s profile?
  - → Part 1: Who are the respondents?
- How do clinicians read radiology CT and MRI reports? Do they read the entire report, and do the different components of a structured report carry the same importance? Is reading behavior influenced by the radiologist’s status (e.g., preliminary reports issued by residents)? What is the perceived value of KI?
  - → Part 2: How do clinicians read the radiology report?
- Once a radiology report is issued, how do clinicians use its content in practice? To what extent are the radiologist’s diagnostic hypotheses and recommendations considered? How are RR incorporated into medical records? Does an emergency context influence clinicians’ attitudes toward RR?
  - → Part 3: What is the use and impact of the radiology report?

Note that although the term « radiology report » was used throughout the manuscript for simplicity and consistency with the literature, respondents were specifically asked about their reading and use of CT and MRI reports.

**Fig. 1:**
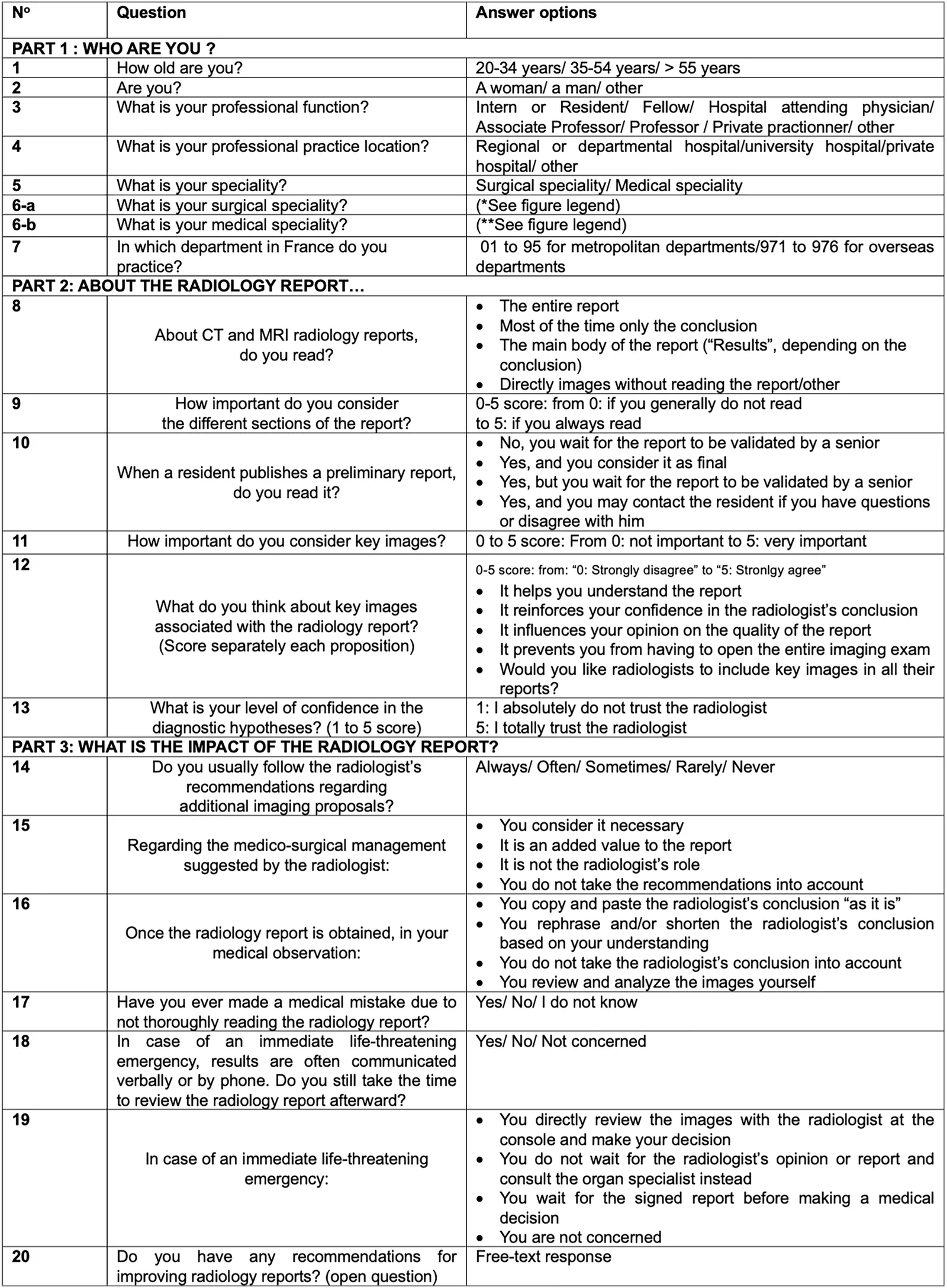
Questionnaire on reading practices of non-radiologists towards RR (CT and MRI). CT, computed tomography; MRI, magnetic resonance imaging. ^*^Maxillofacial surgery/oral surgery/orthopedic and trauma surgery/pediatric surgery/plastic and reconstructive surgery/thoracic and cardiovascular surgery/vascular surgery/visceral and digestive surgery/obstetrics and gynecology/neurosurgery/ophthalmology/otorhinolaryngology (ENT surgery)/urology. ^**^Allergology/anatomical and cytopathology/anesthesia and critical care/medical biology/dermatology/endocrinology/nutrition and diabetology/genetics/geriatrics/medical gynecology/hematology/hepato-gastroenterology/infectious and tropical diseases/cardiovascular medicine/general medicine/intensive care medicine/internal medicine and clinical immunology/forensic medicine and medical expertise/nuclear medicine/physical medicine and rehabilitation/occupational medicine/sport medicine/emergency medicine/vascular medicine/nephrology/neurology/oncology/pediatrics/pneumology/psychiatry/radiology/rheumatology/public health

The questionnaire was therefore divided into three main sections (see Fig. 1 for the detailed questionnaire).

1. Respondent profile (eight questions):
  This section collected information on the respondents’ age, gender, professional status, practice setting (public vs private), geographic region of practice, and medical or surgical specialty.
2. Reading RR (five questions):
  Questions focused on CT and MRI reports’ reading habits (full report, only the body or conclusion, etc.), the importance placed on different sections of the report (i.e.: Study title, Indication (clinical context and referring physician’s question justifying the imaging examination), technique, findings, conclusion, radiologist’s name) and clinicians’ attitudes toward preliminary reports (i.e., the RR edited by radiology residents, before the double reading and validation by a senior radiologist). The impact of KI on understanding the report was also explored.
3. Use of RR and emergency behavior (seven questions): This section aimed to assess clinicians’ confidence in the radiologists’ diagnostic hypotheses, whether they followed recommendations for additional exams suggested, and how the report’s conclusions were integrated into their medical notes. Questions also addressed clinician behavior in cases of immediate life-threatening emergencies and potential medical errors.

A free-text section allowed respondents to provide comments and suggestions for improving imaging reports.

Statistical types of variables collected were:

- Nominal categorical variable: question no. 7
- Ordinal categorical variables: questions no. 1, 2, 3, 4, 5, 6, 8, 9, 14, 15, 16, 17, 18, 19
- Ordinal scale variable: scales of importance/agreement from 0 to 5: questions no. 9, 11, 12; confidence index Likert-type from 1 to 5: question no. 13
- Qualitative textual variable: question no. 20
  The categories were the following:
- Demographic: questions no. 1–7
- Attitudinal (self-reported practice): questions no. 8, 10, 14, 16, 17, 18, 19
- Perceptive (opinion): questions no. 9, 11, 12, 13, 15, 20

### Data analysis

Qualitative variables were summarized as percentages and compared using Chi-square tests. Numerical variables were analyzed with Mann–Whitney *U*-tests or Kruskal–Wallis tests, as appropriate. Effect sizes were assessed with Cramérs’-*V* for categorical variables, and Rank-Biserial correlation for numeric variables. Multivariate regression analyses assessed associations between responses and clinician characteristics (age category, gender, experience, academic status, medical *vs*. surgical status). Analyses were performed in RStudio (2024.12.0), using two-tailed tests, with *p* < 0.05 considered significant.

## Results

### Respondents’ profile

One thousand two hundred fifty-three physicians responded to the questionnaire (response rate = 7.4%), with a mean response time for completing the survey of 5 min and 13 s. See Table 1 for respondents’ characteristics. Most clinicians were aged between 20 and 34 years (71%), and 58% were women. Junior physicians (e.g. interns and residents) were 53.4%, and senior physicians (e.g. fellows, general and specialist medical practitioners) were 46.5%. Academic clinicians were 59.5%, and most respondents were medical specialists (85%), with the majority being general practitioners (24%), emergency medicine practitioners (7.9%), and anesthesiologists/intensivists (6.8%) (Supplemental Fig. 1A*)*. Fifteen percent of respondents came from surgical specialties, with most practicing in orthopedics (19.6%), gynecology-obstetrics (16.9%), and ear nose throat (ENT) (13.7%) (Supplemental Fig. 1B). We received the highest number of responses from the Centre-Val-de-Loire region (*n* = 405), the Parisian region (*n* = 139), and Normandy (*n* = 119), see Supplemental Fig. 2.

**Table 1:** Characteristics of respondents

Category	Total *n* = 1253	Women *n* = 727 (58)	Men *n* = 524 (42)
Age category			
Between 20 and 34 years	883 (70.5)	543 (61.5)	339 (38.4)
Between 35 and 54 years	278 (22.2)	151 (54.3)	126 (45.3)
55 years and more	92 (7.3)	33 (35.9)	59 (64.1)
Status			
Intern or resident	670 (53.5)	409 (61.0)	261 (39.0)
Fellow	90 (7.2)	55 (61.1)	35 (38.9)
Hospital attending practitioner	262 (20.9)	162 (61.8)	100 (38.2)
Private practitioner	180 (14.4)	82 (45.6)	98 (54.4)
University practitioner (professor and associate professor)	37 (3.0)	8(21.6)	29 (78.4)
Other	14 (1.1)	11(78.6)	3 (21.4)
Medical speciality			
General medicine	306 (24.4)	202 (66.0)	104 (34.0)
Emergency medicine	99 (7.9)	58 (58.6)	40 (40.4)
Anesthesiology	85 (6.8)	45 (52.9)	40 (47.1)
Pediatrics	71 (5.7)	55 (77.5)	16 (22.5)
Oncology	70 (5.6)	39 (55.7)	31 (44.3)
Neurology	42 (3.4)	26 (61.9)	16 (38.1)
Cardiology	33 (2.6)	11 (33.3)	22 (66.7)
Rheumatology	30 (2.4)	25 (83.3)	5 (16.7)
Psychiatry	28 (2.2)	13 (46.4)	15 (53.6)
Pulmonology	23 (1.8)	10 (43.5)	13 (56.5)
Dermatology	18 (1.4)	16 (88.9)	2 (11.1)
Endocrinology	19 (1.5)	14 (73.7)	5 (26.3)
Surgical speciality			
Orthopedic surgery	37 (3.0)	7 (18.9)	30 (81.1)
Obstetric gynecology	32 (2.6)	10 (31.3)	22 (68.8)
ENT surgery	26 (2.1)	12 (46.2)	13 (50.0)
Digestive and visceral surgery	22 (1.8)	8 (36.4)	14 (63.6)
Ophthalmology	20 (1.6)	9 (45.0)	11 (55.0)
Urology	10 (0.8)	2 (20.0)	8 (80.0)
Neurosurgery	10 (0.8)	1 (10.0)	9 (90.0)
Maxillofacial surgery	9 (0.7)	4 (44.4)	5 (55.6)
Region			
Center, Loire Valley (Orléans, Tours)	380 (30.3)	207 (54.5)	173 (45.5)
Paris region	145 (11.6)	82 (43.5)	63 (56.6)
South region (Marseille, Lyon)	112 (8.9)	60 (53.6)	52 (46.4)
Normandy	106 (8.5)	58 (54.7)	47 (44.3)
West region	72 (5.8)	48 (66.7)	24 (33.3)
Overseas departments	13 (1.0)	8 (61.5)	5 (38.5)

Count (percentage)

### Main results

#### Reading habits

The most frequent reading pattern was to read the Findings based on the conclusion (31.8%), followed by 28.5% who read the entire report (combined: 60.3% reading the whole report), while 17.4% focused exclusively on the conclusion. Meanwhile, 9.9% of respondents preferred to review the entire image set without reading the report, and a smaller group (12.5%) followed situational reading patterns (i.e: images-only or integrality, results or images-only).

**Fig. 2:**
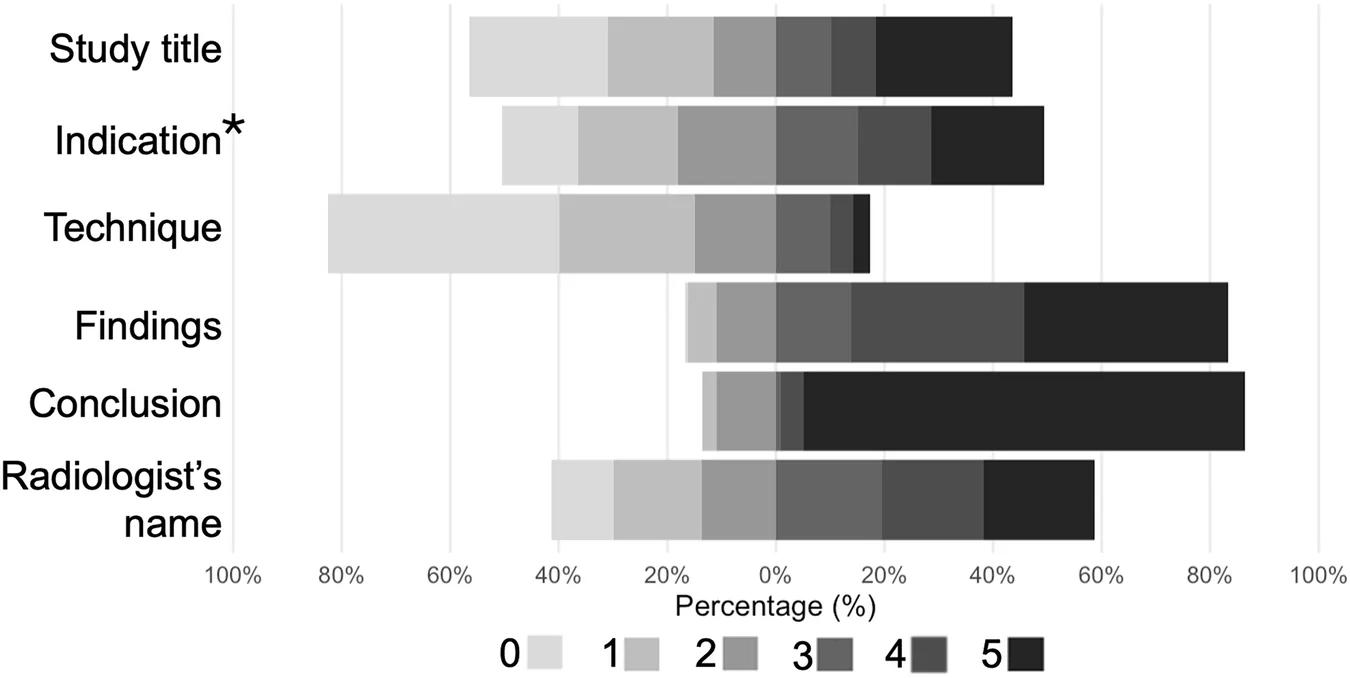
Importance assigned to the different sections of the RR, rated from 0 (generally not read) to 5 (always read). ^*^Indication: summary of the relevant clinical context, including medical history, clinical and laboratory findings, and the specific question addressed to the radiologist by the referring physician, providing the rationale for the imaging examination

Physicians differed in how they value radiology report sections. The conclusion was rated highest (always read, 5/5, for 81%), followed by Findings (always read for 37.51%). The protocol section was often deemed unimportant (42.8% rated 0/5). Clinical indication received moderate importance (13.41% rated 4/5; 20.8% rated 5/5). The radiologist’s name was always read by 20.4% of respondents (details: Fig. 2 and Supplemental Table 1).

#### Preliminary RR

Half of respondents (49.3%) reported reading and considering the report in their medical practice while awaiting final validation by a senior doctor. Additionally, 34.1% of physicians used the preliminary report but contacted the resident for questions, 14.8% regarded the preliminary report as the final version, and 1.7% chose not to use the report until it was formally validated by a senior radiologist.

#### Key Images (KI)

**Fig. 3:**
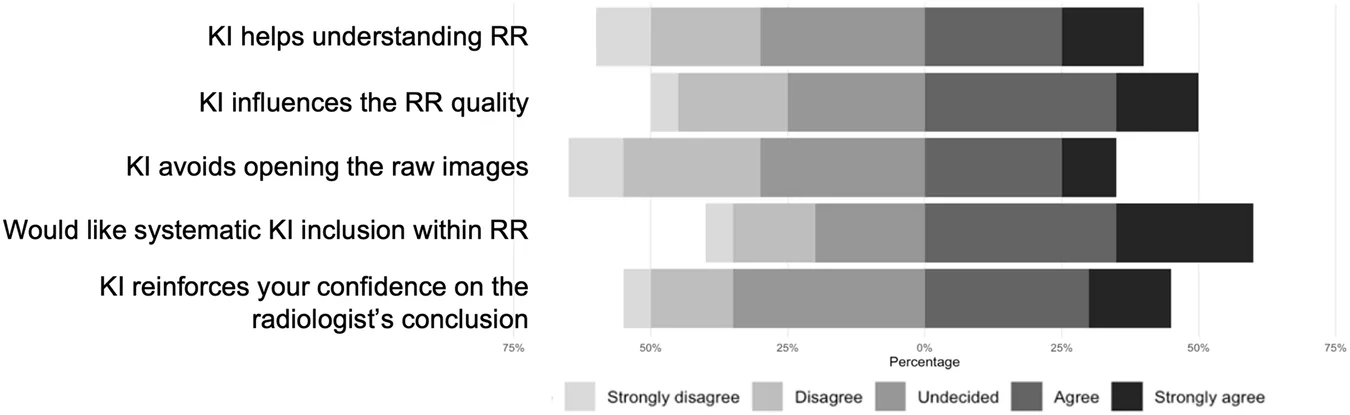
Attitude toward KI (key images, i.e., selected images integrated within the RR)

The average rating score for importance of KI was 3.70 ± 1.18(5-point scale). Figure 3 details attitude towards KI, notably 38.2% of respondents strongly agreed that they aid in understanding the report; 24.5% agreed that they positively influence their perception of the report’s quality. Conversely, 26.9% strongly disagreed with the statement that KI prevents them from reading the report. Moreover, 30.9% of physicians expressed a preference for KI to be systematically included in reports. Finally, 31.6% agreed, and 28.9% strongly agreed that they strengthen their confidence in the radiologist’s conclusion.

#### Recommendations provided by the radiologists

Radiologists’ diagnostic hypotheses inspired relatively high confidence (3.99 ± 0.72/5). Most respondents frequently followed recommendations for additional imaging: 64.6% “often” and 21.8% “always”, while 11.9% did so “sometimes”. Only a small minority “rarely” (1.4%) or “never” (0.2%) adhered to them. Suggestions regarding subsequent medical management were viewed positively by many respondents: 65.6% considered them added value and 7.7% deemed them necessary. However, 21.2% believed such advice lies outside the radiologist’s role, and 5.5% reported not considering these management recommendations.

#### Use and clinical impact of the RR

Most physicians (55.7%) copied the radiologist’s conclusion verbatim, while 39.5% reworded or shortened it; only 4.8% did not take the conclusion into account. About 7% reported having made medical errors due to insufficiently careful reading of RR, whereas 38.8% were unsure and 53.6% denied such errors. In life-threatening emergencies, half of respondents reviewed images directly with the radiologist before deciding, 18.5% contacted the specialist physician directly, and 4.4% waited for the final radiology report before taking action; 27.1% were not concerned by this situation. After a life-threatening emergency, 75.1% reviewed the post-emergency report, 7.7% did not, and 17.2% were not concerned.

#### Results depending on respondents’ profile

**Fig. 4:**
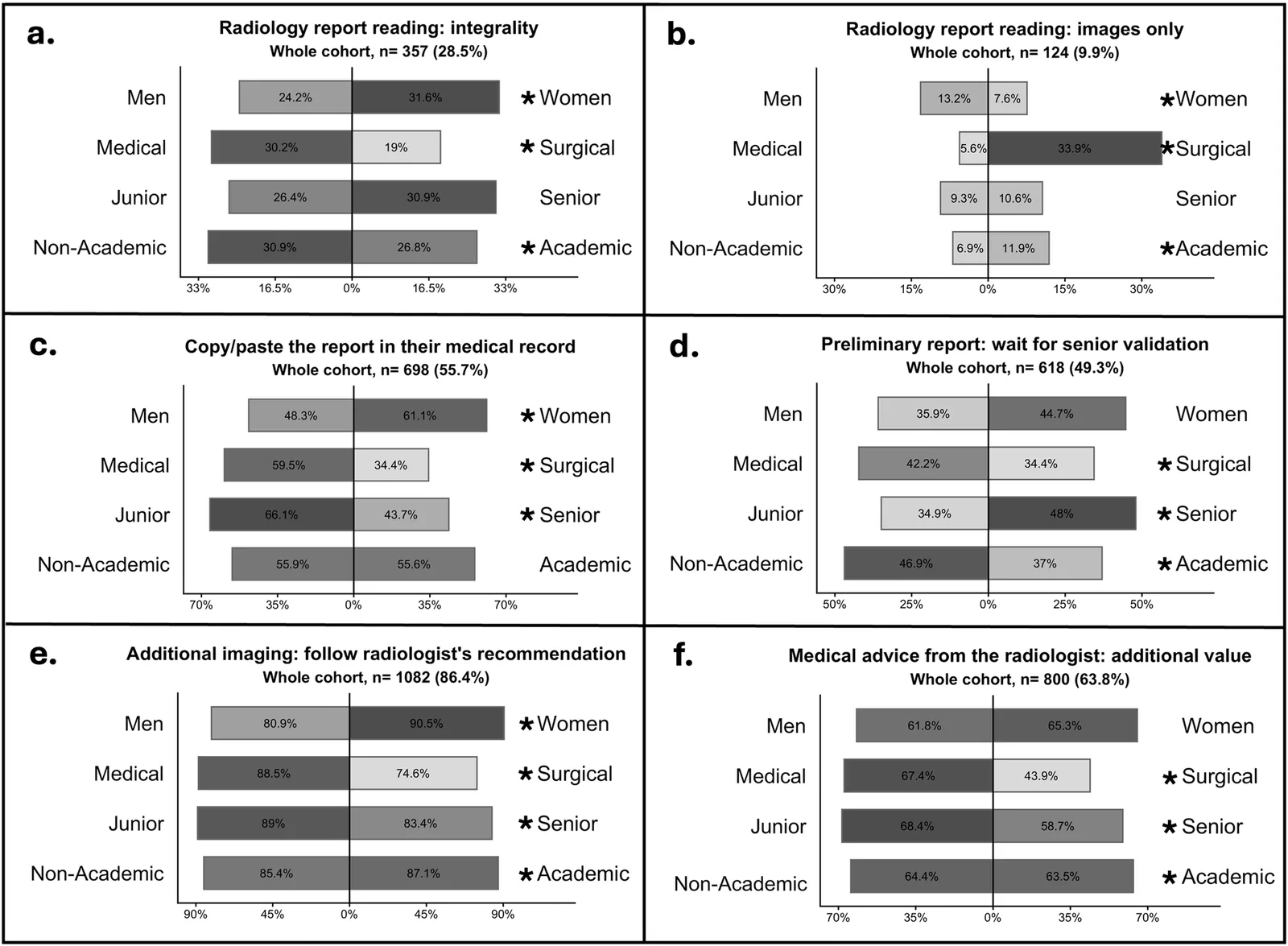
Attitude toward radiology report: summary for main results according to respondents’ profile. Results for the overall cohort (*n* = 1253) for the different parameters are displayed above each horizontal histogram chart. Comparison of four respondent groups: gender (men vs women), subspeciality (medical vs surgical), experience (junior vs senior) and institution (non-academic: regional hospitals and private practice establishments vs academic: university hospitals). Detailed results are provided in Tables 2–4 and in Supplemental Table 3. ^*^Indicates a statistically significant difference combining a *p* value < 0.001 and an effect size > 0.100 in the main analysis. **a** Proportions of respondents declaring that they read the entire radiology report. **b** Proportions of respondents reading images only. **c** Proportions of respondents copy-pasting the radiology report unchanged into the medical record. **d** Attitude toward preliminary reports written by residents: waiting for the senior validation before making a medical decision. **e** Proportion of respondents following the radiologist’s recommendation to perform additional imaging (combination of «always» and «often» responses). **f** Proportion of respondents considering that radiologist’s advice on patient medical management provides additional value

**Fig. 5:**
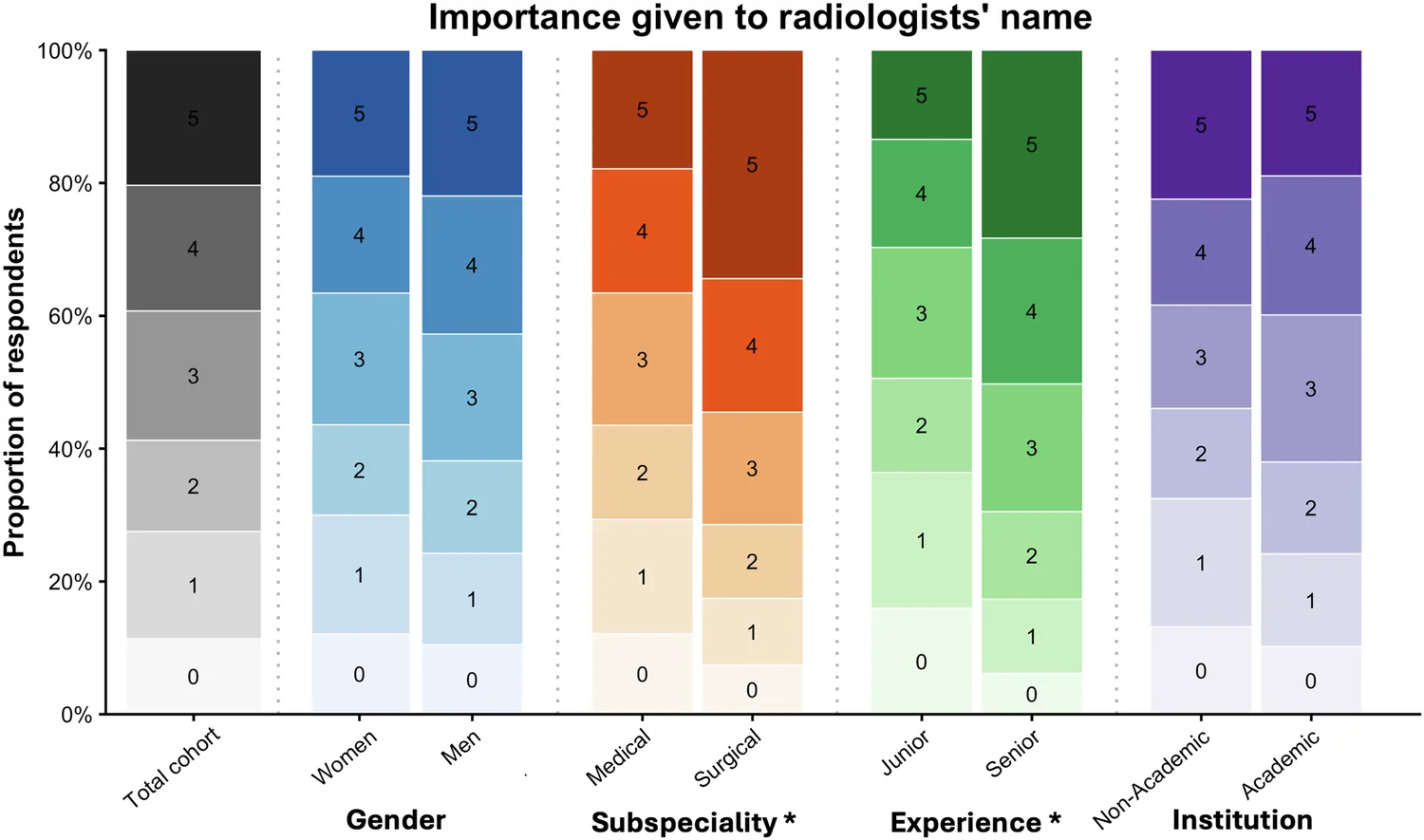
Importance given to the radiologist’s name when reading RR. Five-point score: 0 = the radiologist’s name is never read; 5 = the radiologist’s name is always read. The leftmost gray-scale histogram bar represents the results for the overall cohort. Subgroup results are displayed in color according to the respondent’s profile (gender: blue scale; subspeciality: orange scale; experience: green scale; institution type: purple scale). ^*^Indicates a statistically significant difference combining a *p* value < 0.001 and an effect size > 0.100 in the main analysis

Subgroup analyses based on gender, experience, age, academic status, and medical vs surgical specialties revealed significant but generally small effect-size differences (Tables 2–4 and Supplemental Tables 2 and 3*)*. A few big differences were observed, being more frequent when comparing academic vs non-academic respondents, and strongest when comparing medical vs surgical profiles. Figure 4 summarizes the main differences in results across respondent profiles, whereas Fig. 5 focuses specifically on the importance attributed to the radiologist’s name within these profiles.

**Table 2:** Responses depending on gender (women vs men)

		GENDER			
		Women *n* = 727 (58)	Men *n* = 524 (42)	*p* value	Effect size
RADIOLOGY REPORT reading pattern					
	Integrality	230 (31.6)	127 (24.2)	**< 0.001**	**0.147**
	Results depending on conclusion	244 (33.6)	153 (29.2)	-	
	Conclusion only	123 (16.9)	95 (18.1)	-	
	Images only	55 (7.6)	69 (13.2)	-	
	Situational	75 (10.3)	80 (15.3)		
RADIOLOGY REPORT, Importance given for each section (/5)					
	Title	2.30 ± 2.0	2.33 ± 1.9	0.815	0.007
	Indication	2.51 ± 1.7	2.68 ± 1.7	0.101	0.046
	Technique	1.09 ± 1.3	1.29 ± 1.4	0.001	0.090
	Results	3.86 ± 1.2	3.81 ± 1.2	0.513	0.018
	Conclusion	4.50 ± 1.1	4.51 ± 1.1	0.317	0.028
	Name	2.70 ± 1.7	2.92 ± 1.7	0.021	0.065
Key Images					
	Notation (/5)	3.72 ± 1.2	3.67 ± 1.2	0.648	0.009
Help understanding the radiology report	Strongly agree	292 (40.2)	187 (35.7)	0.572	0.048
	Agree	286 (39.3)	222 (42.3)	-	
	Undecided	95 (13.1)	73 (13.9)	-	
	Disagree	29 (3.9)	25 (3.4)	-	
	Strongly disagree	25 (3.4)	17 (3.2)	-	
Enhance report quality	Strongly agree	101(13.9)	103 (19.7)	**0.002**	**0.116**
	Agree	169 (23.2)	137 (26.2)	-	
	Undecided	274 (37.6)	150 (28.6)	-	
	Disagree	119 (16.3)	76 (14.5)	-	
	Strongly disagree	64 (8.8)	58 (11.1)	-	
Avoid opening the imaging examination	Strongly agree	96 (13.2)	49 (9.3)	0.018	0.098
	Agree	141(19.3)	104 (19.8)	-	
	Undecided	136 (18.7)	89 (16.9)	-	
	Disagree	181 (24.9)	117 (22.3)	-	
	Strongly disagree	173(23.8)	165 (31.4)	-	
Would like their systematic inclusion in radiology report	Strongly agree	243 (33.4)	162 (30.9)	0.232	0.067
	Agree	224 (30.8)	162 (30.9)	-	
	Undecided	180 (24.7)	120 (22.9)	-	
	Disagree	43 (5.9)	46 (8.7)	-	
	Strongly disagree	37 (5.1)	34 (6.4)	-	
Reinforce confidence toward the radiologist’s conclusion	Strongly agree	198 (27.2)	163 (30.1)	0.076	0.082
	Agree	221 (30.4)	174 (33.2)	-	
	Undecided	224 (30.8)	124 (23.6)	-	
	Disagree	54 (7.4)	37 (7.1)	-	
	Strongly disagree	30 (4.1)	26 (4.9)	-	
USE of THE RADIOLOGY REPORT					
Notation of the radiologist’s diagnostic hypothesis (/5)		4.06 ± 0.69	3.91 ± 0.75	**< 0.001**	**0.101**
Do you follow radiologist’s recommendation for additional imaging?	Always	176 (24.2)	97 (18.5)	**< 0.001**	**0.150**
	Often	482 (66.3)	327 (62.4)		
	Sometimes	61 (8.3)	87 (16.6)		
	Rarely	8 (1.1)	10 (1.9)		
	Never	0	3 (0.5)		
How do you consider the radiologist’s medical care recommendation?	It is necessary	27 (3.7)	14 (2.6)	< 0.001	0.094
	It has an additional value	475 (65.3)	324 (61.8)		
	Not his/her role	118 (16.2)	86 (16.4)		
	Do not follow	29 (3.9)	19 (3.6)		
Use of the RR within the medical record	Copy/paste	444 (61.1)	253 (48.2)	**< 0.001**	**0.166**
	Reformulate	266 (36.5)	228 (43.5)		
	Do not consider	17(2.4)	43 (8.2)		
Have you ever made a mistake by not reading the RR?	Yes	42 (5.7)	53 (10.1)	< 0.001	0.009
	No	389 (53.5)	283 (54.1)		
	Do not know	296(40.7)	188 (35.8)		
About the preliminary report, do you read it?	Yes, consider it definitive	87 (11.9)	73 (13.9)	< 0.001	0.003
	Yes, but wait for senior validation	325 (44.7)	188 (35.8)		
	Yes, but call if disagree	124 (17.1)	121 (23.1)		
	No, wait for senior validation	11 (1.51)	16 (3.1)		
	Not concerned	4 (0.5)	10 (1.9)		
In a vital emergency, what is your attitude toward the RR	You watch images directly	334 (45.9)	290 (55.3)	< 0.001	0.013
	Do not wait, ask a specialist to watch	145 (19.9)	87 (16.6)		
	Wait final RR	35 (4.8)	20 (3.8)		
	Not concerned	213 (29.3)	127 (24.2)		
Do you read the RR post-emergency?	Yes	554 (76.07)	386 (73.6)	0.065	0.066
	No	45 (6.19)	51 (9.7)		
	Not concerned	129 (17.74)	87 (16.6)		

Data are count (percentage) or mean ± standard deviation. Bolded *p* values and effect size results indicate an effect size > 0.100

**Table 3:** Responses depending on subspecialty (medical vs surgical)

		SPECIALITY			
		Medical *n* = 1064 (85)	Surgical *n* = 189 (15)	*p* value	Effect size
RADIOLOGY REPORT reading pattern					
	Integrality	321 (30.2)	36 (19.1)	**< 0.001**	**0.345**
	Results depending on conclusion	363 (34.1)	35 (18.5)	-	
	Conclusion only	189 (17.8)	29 (15.3)	-	
	Images only	60 (5.6)	64 (33.9)	-	
	Situational	131 (12.3)	25 (13.2)		
RADIOLOGY REPORT, importance given for each section (/5)					
	Title	2.32 ± 1.9	2.23 ± 2.0	< 0.001	0.020
	Indication	2.57 ± 1.7	2.64 ± 1.8	< 0.001	0.016
	Technique	1.16 ± 1.3	1.21 ± 1.5	< 0.001	0.004
	Results	3.87 ± 1.19	3.66 ± 1.34	< 0.001	0.048
	Conclusion	4.54 ± 1.10	4.32 ± 1.21	**< 0.001**	**0.115**
	Name	2.69 ± 1.65	3.35 ± 1.63	**< 0.001**	**0.146**
Key Images					
	Notation (/5)	3.71 ± 1.2	3.63 ± 1.2	0.504	0.019
Help understanding the radiology report	Strongly agree	422 (39.6)	57 (30.1)	< 0.001	0.081
	Agree	427 (40.1)	82 (43.3)		
	Undecided	140 (13.1)	29 (15.3)		
	Disagree	42 (3.9)	12 (6.3)		
	Strongly disagree	33 (3.1)	9 (4.7)		
Enhance report quality	Strongly agree	164 (15.4)	41 (21.6)	< 0.001	0.071
	Agree	262 (24.6)	45 (23.8)		
	Undecided	365 (34.3)	59 (31.2)		
	Disagree	164 (15.4)	31 (16.4)		
	Strongly disagree	109 (10.2)	13 (6.8)		
Avoid opening the imaging examination	Strongly agree	133 (12.5)	12 (6.3)	**< 0.001**	**0.205**
	Agree	234 (21.9)	13 (6.8)		
	Undecided	189 (17.7)	36 (19.1)		
	Disagree	256 (24.1)	42 (22.2)		
	Strongly disagree	252 (23.6)	86 (45.5)		
Would like their systematic inclusion in radiology report	Strongly agree	343 (32.2)	62 (32.8)	< 0.001	0.060
	Agree	337 (31.6)	51 (26.9)		
	Undecided	256 (24.1)	44 (23.2)		
	Disagree	70 (6.5)	19 (10.1)		
	Strongly disagree	58 (5.4)	13 (6.8)		
Reinforce confidence toward the radiologist’s conclusion	Strongly agree	304 (28.5)	58 (30.6)	< 0.001	0.049
	Agree	346 (32.5)	50 (26.4)		
	Undecided	290 (27.2)	58 (30.6)		
	Disagree	76 (7.1)	15 (7.9)		
	Strongly disagree	48 (4.5)	8 (4.2)		
USE of THE RADIOLOGY REPORT					
Notation of the radiologist’s diagnostic hypothesis (/5)		4.06 ± 0.68	3.62 ± 0.83	**< 0.001**	**0.233**
Do you follow radiologist’s recommendation for additional imaging?	Always	256 (24.1)	17 (9.0)	**< 0.001**	**0.219**
	Often	686 (64.5)	124 (65.6)		
	Sometimes	113 (10.6)	36 (19.0)		
	Rarely	9 (0.8)	9 (4.8)		
	Never	0	3 (1.6)		
How do you consider the radiologist’s medical care recommendation?	It is necessary	36 (3.4)	5 (2.6)	**< 0.001**	**0.267**
	It has an additional value	717 (67.4)	83 (43.9)		
	Not his/her role	153 (14.4)	52 (27.5)		
	Do not follow	29 (2.7)	19 (10.1)		
Use of the RR within the medical record	Copy/Paste	633 (59.5)	65 (34.4)	**< 0.001**	**0.321**
	Reformulate	409 (38.4)	86 (45.5)		
	Do not consider	22 (2.1)	38 (20.1)		
Have you ever made a mistake by not reading the RR?	Yes	81 (7.6)	14 (7.4)	< 0.001	0.012
	No	568 (53.4)	104 (55.0)		
	Do not know	415 (39.0)	71 (37.6)		
About the preliminary report, do you read it?	Yes, consider it definitive	146 (13.7)	15 (7.9)	**< 0.001**	**0.237**
	Yes, but wait for senior validation	449 (42.1)	65 (34.3)		
	Yes, but call if disagree	184 (17.2)	61 (32.2)		
	No, wait for senior validation	15 (1.4)	12 (6.3)		
	Not concerned	6 (0.5)	8 (4.2)		
In a vital emergency, what is your attitude toward the RR	You watch images directly	509 (47.8)	117 (61.9)	**< 0.001**	**0.134**
	Do not wait; ask a specialist to watch	216 (20.3)	16 (8.4)		
	Wait final RR	52 (4.8)	3 (1.5)		
	Not concerned	287 (26.9)	53 (28.1)		
Do you read the RR post-emergency?	Yes	825 (77.5)	116 (61.3)	**< 0.001**	**0.163**
	No	64 (6.1)	32 (16.9)		
	Not concerned	175 (16.4)	41 (21.6)		

Data are count (percentage) or mean ± standard deviation. Bolded *p* values and effect size results indicate an effect size > 0.100

**Table 4:** Responses depending on experience (junior vs senior)

		EXPERIENCE			
		Junior *n* = 670 (53.4)	Senior *n* = 583 (46.5)	*p* value	Effect size
RADIOLOGY REPORT reading pattern					
	Integrality	177 (26.4)	180 (30.9)	0.090	0.080
	Results depending on conclusion	235 (35.1)	163 (28.0)	-	
	Conclusion only	116 (17.3)	102 (17.5)	-	
	Images only	62 (9.3)	62 (10.6)	-	
	Situational	80 (11.9)	76 (13.0)		
RADIOLOGY REPORT, importance given for each section (/5)					
	Title	2.08 ± 1.9	2.58 ± 1.9	**< 0.001**	**0.132**
	Indication	2.46 ± 1.7	2.71 ± 1.7	< 0.001	0.072
	Technique	0.91 ± 1.3	1.47 ± 1.4	**< 0.001**	**0.237**
	Results	3.73 ± 1.2	3.96 ± 1.2	**< 0.001**	**0.101**
	Conclusion	4.46 ± 1.2	4.55 ± 1.1	< 0.001	0.024
	Name	2.40 ± 1.7	3.24 ± 1.6	**< 0.001**	**0.253**
Key Images					
	Notation (/5)	3.69 ± 1.2	3.70 ± 1.2	0.493	0.019
Help understanding the radiology report	Strongly agree	254 (37.9)	225 (38.6)	0.034	0.091
	Agree	291 (43.4)	218 (37.3)	-	
	Undecided	86 (12.8)	83 (14.2)	-	
	Disagree	24 (3.5)	30 (5.2)	-	
	Strongly disagree	15 (2.2)	27 (4.6)	-	
Enhance report quality	Strongly agree	109 (16.3)	96 (16.5)	0.191	0.070
	Agree	167 (24.9)	140 (24.0)	-	
	Undecided	220 (32.8)	204 (35.0)	-	
	Disagree	117 (17.5)	78 (13.4)	-	
	Strongly disagree	57 (8.5)	65 (11.2)	-	
Avoid opening the imaging examination	Strongly agree	72 (10.8)	73 (12.5)	0.240	0.066
	Agree	138 (20.6)	109 (18.7)	-	
	Undecided	117 (17.5)	108 (18.5)	-	
	Disagree	173 (25.8)	125 (21.4)	-	
	Strongly disagree	170 (25.4)	168 (28.8)	-	
Would like their systematic inclusion in radiology report	Strongly agree	213 (31.7)	192 (32.9)	**0.006**	**0.108**
	Agree	213 (31.7)	175 (30.1)	-	
	Undecided	175 (26.1)	125 (21.4)	-	
	Disagree	45 (6.7)	44 (7.5)	-	
	Strongly disagree	24 (3.5)	47 (8.1)	-	
Reinforce confidence toward the radiologist’s conclusion	Strongly agree	208 (31.1)	154 (26.4)	0.067	0.084
	Agree	205 (30.6)	191 (32.7)	-	
	Undecided	185 (27.6)	163 (27.9)	-	
	Disagree	51 (7.6)	40 (6.8)	-	
	Strongly disagree	21 (3.1)	35 (6.1)	-	
USE of THE RADIOLOGY REPORT					
Notation of the radiologist’s diagnostic hypothesis (/5)		4.03 ± 0.7	3.95 ± 0.8	0.186	0.070
Do you follow radiologist’s recommendation for additional imaging?	Always	131 (19.6)	142 (24.4)	**< 0.001**	**0.131**
	Often	465 (69.4)	345 (59.2)		
	Sometimes	70 (10.5)	79 (13.6)		
	Rarely	4 (0.6)	14 (2.4)		
	Never	0	3 (0.5)		
How do you consider the radiologist’s medical care recommendation?	It is necessary	25 (3.7)	16 (2.7)	**< 0.001**	**0.149**
	It has an additional value	458 (68.3)	342 (58.6)		
	Not his/her role	82 (12.2)	123 (21.1)		
	Do not follow	20 (2.9)	28 (4.8)		
Use of the RR within the medical record	Copy/Paste	443 (66.1)	255 (43.7)	**< 0.001**	**0.232**
	Reformulate	210 (31.3)	285 (48.8)		
	Do not consider	17 (2.5)	43 (7.3)		
Have you ever made a mistake by not reading the RR?	Yes	45 (6.7)	50 (8.6)	0.458	0.025
	No	364 (54.3)	308 (52.8)		
	Do not know	261 (39.0)	225 (38.6)		
About the preliminary report, do you read it?	Yes, consider it definitive	102 (15.2)	59 (10.1)	**< 0.001**	**0.226**
	Yes, but wait for senior validation	234 (34.9)	280 (48.1)		
	Yes, but call if disagree	146 (21.7)	99 (16.9)		
	No, wait for senior validation	5 (0.7)	22 (3.7)		
	Not concerned	0	14 (2.4)		
In a vital emergency, what is your attitude toward the RR	You watch images directly	340 (50.7)	286 (49.1)	< 0.001	0.075
	Do not wait; ask a specialist to watch	134 (20.1)	98 (16.8)		
	Wait final RR	33 (4.9)	22 (3.7)		
	Not concerned	163 (24.3)	177 (30.3)		
Do you read the RR post-emergency?	Yes	529 (78.9)	412 (70.6)	< 0.001	0.096
	No	43 (6.4)	53 (9.1)		
	Not concerned	98 (14.6)	118 (20.2)		

Data are counts (percentage) or mean ± standard deviation. Bolded *p* values and effect size results indicate an effect size > 0.100

#### Gender (Table 2)

Women tended more frequently to read the RR integrity and to copy/paste the conclusion into their medical records compared to men. Women were more frequently undecided about the fact that KI enhances RR reporting.

#### Medical vs surgical profile (Table 3)

Surgeons more frequently reported reading only the images (surgeons: 28.0% vs medical-specialist: 5.2%, *p* < 0.001, Cramers’-*V* = 0.353) and were more likely to disregard the radiology report (20.1% vs 2.1%) or reformulate its conclusion 45.5% vs 38.4%, *p* < 0.001, Cramers’-*V* = 0.321). Medical clinicians rated radiologists’ diagnostic hypotheses more favorably. Surgeons were less convinced that KI reduces the need to open the full exam, assigned greater importance to the radiologist’s name, and more often viewed management recommendations as outside the radiologist’s role. They more commonly read preliminary reports but contacted senior radiologists when disagreeing, and tended to follow additional imaging recommendations only sometimes, whereas medical clinicians did so more consistently.

#### Experience (Table 4)

Seniors rated all RR items higher, particularly the imaging protocol and radiologist’s name. Juniors more often copied the conclusion, whereas seniors tended to reformulate it and were more likely to wait for the final report before deciding. Age and experience showed similar trends; differences based on the 3 age groups are displayed in Supplemental Table 2.

#### Academic profile (Supplemental Table 3)

Academic practitioners rated KI more highly, and more often agreed they help substitute for opening images and improve understanding of the report. Non-academic clinicians showed higher confidence in radiologists’ diagnostic hypotheses and more frequently waited for the final senior report. In emergencies, academics more often directly reviewed images, while non-academics were more often not concerned by this scenario.

#### Medical subspecialities (Supplemental Table 4)

Differences were observed across medical subspecialities. General practitioners more frequently reported reading the Results sections in conjunction with the Conclusion (46.7% vs 34.1% in the overall medical group, *p* < 0.001). In contrast, emergency physicians were less likely to read the entire report (20.2% vs 30.2%, *p* < 0.001) and more frequently copy/pasted the radiologist’s conclusion (88.9% vs 59.5%, *p* < 0.001). Conversely, oncologists more often reformulated the report (58.0% vs 38.4%, *p* < 0.001).

Regarding preliminary reports issued by residents, cardiologists, pediatricians, general practitioners or oncologists more frequently waited for a senior radiologist’s validation before making a clinical decision, whereas emergency physicians were more likely to consider the preliminary report as definitive.

### Open suggestions

Two hundred ninety-one respondents (23.2%) provided free-text suggestions (51.8% women, 65% senior doctors, 33.7% academics). Comments highlighted recurrent needs: shorter, clearer conclusions; visual emphasis on key information; systematic inclusion of KI for clarity and teaching; and immediate clinician notification when preliminary reports are significantly revised. Concerning oncology imaging, systematic inclusion of tumor node metastasis (TNM) classifications and response evaluation criteria in solid tumors scores was requested.

## Discussion

The perception and use of RR by referring physicians have garnered increasing attention but remain sparsely explored. In this nationwide multicenter survey, we adopted the perspective of referring clinicians and characterized how RRs are read and integrated into routine practice, focusing on CT and MRI reports. The Findings and Conclusion sections were the most valued, whereas attention to the radiologist’s name varied across profiles. Key images were particularly valued by academic practitioners and tended to increase confidence in radiologic interpretations. Reading patterns differed notably between medical and surgical specialists and, to a lesser extent, between academic and non-academic clinicians, highlighting the need to adapt reporting practices to diverse user expectations.

Unlike studies limited to specific subspecialties [20, 26], academic status [25], or single centers [24], our broad inclusion criteria aimed for a more representative sample. The female predominance (58%) partially reflects current medical demographics in France, where women account for 50% of practitioners [27]. This contrasts with prior surveys with a smaller female proportion (26–36.5% [19, 25]), offering a more balanced sample, unlike studies that did not report gender distribution [24].

The respondent population was predominantly young (< 35 years, 71%), a group spanning diverse professional stages from interns to early-career senior specialists. This diversity captures physicians who are actively engaged with RR and familiar with their clinical use. While finer age stratification could have added granularity, collecting medical status enabled differentiation of experience levels. As these younger physicians will shape future clinical practice, their strong participation provides valuable insight into current and emerging patterns of RR use.

Our sample included many physicians from medical specialties (85%), consistent with national demographics but leading to underrepresentation of surgeons, limiting analysis of their specific behaviors. Targeted surveys of surgical groups may therefore be more informative. Previous work reported that 20% of orthopedic surgeons never consult plain radiograph reports, 4% neglect CT reports, and none disregard MRI reports [20]. Our nationwide multicenter design intended to reflect routine practice, contrasting with Kwee et al’s academically oriented sample, which may limit the generalizability of their findings [25].

A survey to evaluate how physicians truly read RR was the most straightforward approach. Despite reliance on self-reporting, responses likely reflect actual practices, whereas a real-life assessment such as eye-tracking would be complex and could alter reader behavior.

Except for surgeons, who more often rely on imaging alone, reading practices did not vary markedly across physician profiles. Most clinicians read the full report or its conclusion, underscoring the RR's importance.

The 7% who reported an error due to insufficient reading further highlight the RR’s critical role in care.

The radiologist’s signature is important, particularly among senior clinicians. Most prior studies did not examine this aspect [18, 24, 25], though Iyengar et al included the radiologist’s name and grade as key elements [28]. This finding underscores the importance of radiologists’ experience and local trust networks, while suggesting that the anonymity of external teleradiology may feel less reliable, reinforcing the need to strengthen clinician-radiologist trust. We excluded teleradiology practices, as it represents a separate topic whose inclusion would introduce many additional questions and overly lengthen the questionnaire.

Clinico-radiological correlation is essential both for justifying imaging examination and for ensuring the appropriate interpretation of radiological findings. In our study, the Indication section was read heterogeneously by respondents: approximately half considered it an important component of the report, whereas the remainder tended to pay limited attention to it. The Indication section is essential not only because it provides the clinical context necessary for image interpretation, but also because it helps ensure that the requested examination is justified, appropriate, and clinically relevant. We did not further explore radio-clinical correlation in the present study. Assessing the pertinence and clinical impact of imaging examinations requires a dedicated analysis integrating multiple parameters, including the clinical presentation, anatomical region, imaging modality, and radiological findings and conclusion. Such an approach was beyond the scope of the current work

The inclusion of KI has been previously suggested [8, 10–12], and our findings confirm their substantial value. KI improve report comprehension, support education for seniors and trainees, and strengthen trust in the radiologist’s conclusion. Yet their use remains limited, likely due to the added time required for integration. This raises the question of whether future artificial intelligence tools could facilitate the rapid and systematic incorporation of KI, thereby improving efficiency and accessibility.

The way non-radiologists integrate RR into their medical record has been insufficiently explored. This behavior varied across respondent profiles, although copy-paste use is more frequent, except for seniors and surgeons. While some surgeons and physicians primarily review images, diagnostic hypotheses remain a key component of reports. Despite relatively low discrepancy rates between resident reports and senior reports, influenced by academic level and clinical context [29*–*31], half of respondents considered preliminary reports while awaiting senior validation, reflecting a cautious approach and underscoring the need to minimize delays between preliminary and final reports. In emergencies, most clinicians revisit the report afterward, underscoring its critical role.

Although reading patterns differed mainly between medical and surgical specialists and, to a lesser extent, academic and non-academic profiles, several gender-related trends emerged, but without significant effect sizes. Women more often read the entire report and copy the conclusion, suggesting a more meticulous approach, whereas men more frequently reformulate or disregard it.

Analysis of medical subspecialties revealed notable differences. Emergency physicians were more likely to copy/paste the radiologist’s conclusion, whereas oncologists more frequently reformulated the report content. This original finding further supports the notion that the readers’ profiles are heterogeneous and highlights the importance of conducting specialty-specific analyses of the different specialties in future studies.

Among open comments, the need for clearer conclusions is in line with reporting preferences highlighted by Kowalczyk et al, who showed that the blended report was preferred over a narrative or highly templated report [21], and a literature review [22]. The emphasis on visually highlighting key information and incorporating KI aligns with our main findings.

Our study has limitations. Despite a large sample, responses were unevenly distributed across France, largely due to dissemination through social media, personal networks, and local professional contact. Future surveys should use national or international institutional mailing lists to achieve a more balanced geographical representation. The survey was distributed between 2023 and 2024, so the data are no longer fully current. Still, from a broader temporal perspective, these data remain relevant, and the temporal distance may itself be valuable. Our work could serve as a basis for future similar surveys and allow longitudinal assessment of radiology report reading habits over time. The growing role of externalized teleradiology may represent an additional aspect to be explored in future questionnaires or could itself become the subject of a dedicated survey. Our sample overrepresented younger practitioners, potentially limiting senior representation, though differences were not consistently significant and younger respondents reflect the future cohort of experienced clinicians. Self-reporting bias is possible, as clinicians may overestimate reading rigor. Additionally, interpretation of open-ended responses is subjective and less reproducible.

Although the respondents were informed that the questionnaire specifically addressed CT and MRI reports, their responses may not have been restricted exclusively to these two imaging modalities. Previous studies have shown that reading habits may vary according to the type of imaging examination [20]. Therefore, our findings may not fully capture the nuances in clinicians’ attention given to the different imaging modalities. Furthermore, reading behavior is likely to be highly context-dependent. For a given physician, the way a radiology report is read may depend not only on the imaging modality but also on several other factors, such as the clinical presentation of the patient, the time of the day, workload, and the clinician’s cognitive state at the time of interpretation.

As a self-reported survey, our study cannot capture all of these situational nuances. Nevertheless, it provides a valuable overall framework for understanding how clinicians read and use RR in routine practice.

Given the expanding role of artificial intelligence, large language models (LLMs) have the potential to automate and enhance structured RR [25], although adoption remains limited by their rigidity and implementation demands. By transforming free text, assisting documentation, and reducing textual errors, LLMs could support tasks such as automatic TNM classification, RadGPT terms generation, or organizing KI within reports [32]. The survey we propose may serve as a reference for future studies involving larger cohorts or international comparisons of reporting habits.

## Conclusion

Optimizing RR requires understanding how referring clinicians read and use them. This survey identified consistent patterns: the Findings and Conclusion sections were most valued, while attention to the radiologist’s name varied by clinician profile. Key images were especially valued by academic practitioners and generally strengthened confidence in radiologists’ conclusions, supporting discussion of their routine inclusion. Report conclusions were largely incorporated into records, either directly or after reformulation. A non-negligible rate of reading-related errors was identified. Notable interpretation differences emerged primarily between medical and surgical clinicians, and to a lesser extent between academic and non-academic practitioners. Future studies should explore these dynamics internationally and longitudinally.

## Supplementary information

ELECTRONIC SUPPLEMENTARY MATERIAL

## Data Availability

Data are available upon reasonable request from the authors.

## Abbreviations

ENT: Ear nose throat
KI: Key images
RR: Radiology reports
TNM: Tumor node metastasis
