# Supplementary material for: How do non-radiologist physicians read and use the radiology report? A French national survey
Source: Insights Imaging. 2026 Sep 5;17:222. doi: 10.1186/s13244-026-02387-1 (PMC13546390; doi:10.1186/s13244-026-02387-1)
Supplement: Supplementary file 1 [file 13244_2026_2387_MOESM1_ESM.pdf]

How do non-radiologists physicians read and use the radiology report? A French national survey

ELECTRONIC SUPPLEMENTARY MATERIAL

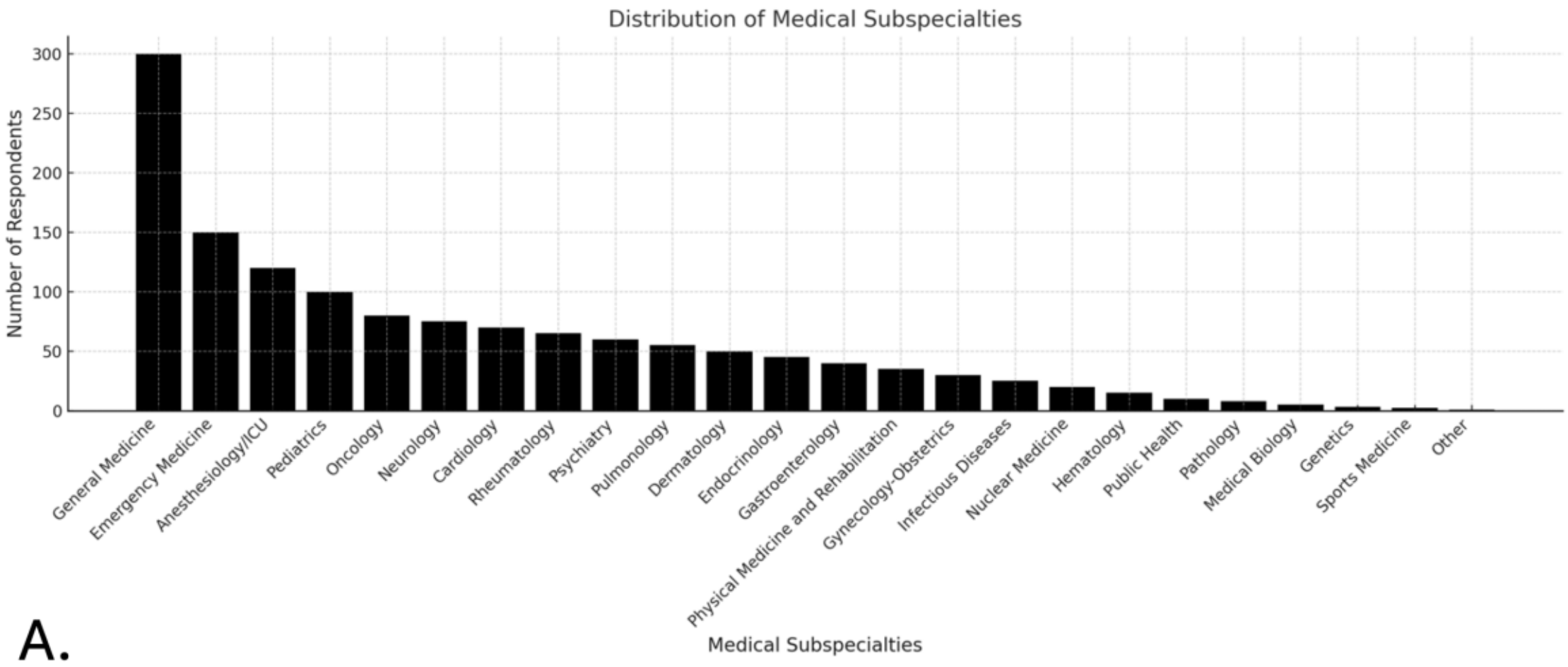

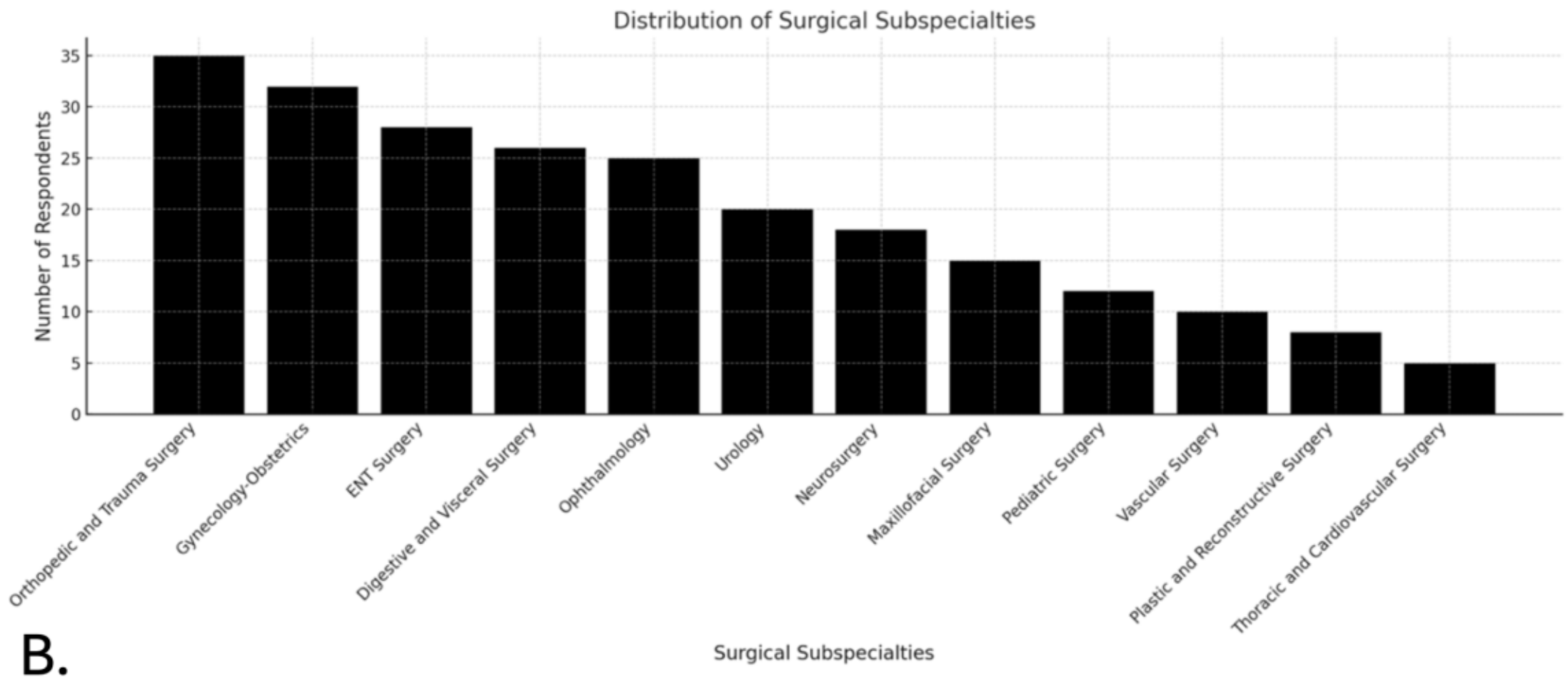

Supplemental Figure 1. Distribution of Medical (A) and Surgical (B) Subspecialties

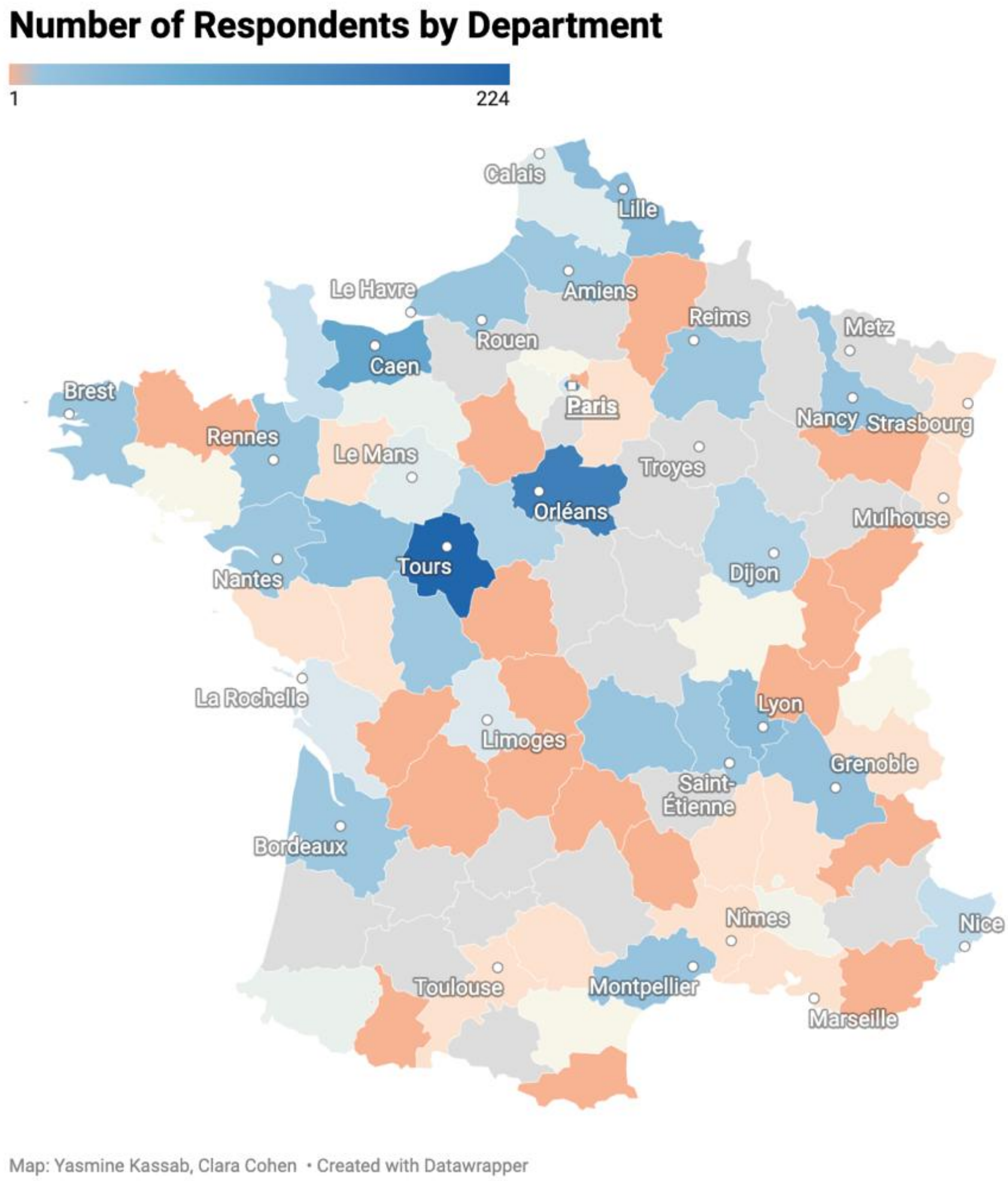

Supplemental Figure 2. Number of Respondents by Department
(interactive link : https://datawrapper.dwcdn.net/aC7QT/1/)

Rating	Study title	Indication	Procedure Type	Findings	Conclusion	Radiologist's Name
**0**	320 (25.5)	176 (14.1)	536 (42.8)	6 (0.5)	1 (0.1)	143 (11.4)
**1**	244 (19.5)	232 (18.5)	313 (25.0)	67 (5.4)	33 (2.6)	202 (16.1)
**2**	144 (11.5)	225 (18.0)	186 (14.8)	136 (10.9)	136 (10.9)	172 (13.7)
**3**	129 (10.3)	191 (15.2)	127 (10.1)	174 (13.9)	12 (1.0)	244 (19.5)
**4**	103 (8.2)	168 (13.4)	52 (4.2)	400 (31.9)	53 (4.2)	237 (18.9)
**5**	313 (25.0)	261 (20.8)	39 (3.1)	470 (37.5)	1018 (81.3)	255 (20.4)

Supplemental Table 1. Importance given to each section of the RR (from 0 (generally not read) to 5/5 (always read)) – Number of respondents and proportions

		AGE CATEGORY				
		**20-34 years n=885 (70.6)**	**35-54 years n=276 (22.1)**	***>55 years* n=92 (7.3)**	***P* value**	**Effect Size**
**RADIOLOGY REPORT Reading pattern**						
	Integrality	239 (27.0)	79 (28.7)	39 (42.4)	**<0.001**	**0.114**
	Results depending on conclusion	307 (34.7)	74 (26.8)	17 (18.5)	-	
	Conclusion only	161 (18.2)	42 (15.1)	15 (16.3)	-	
	Images only	76 (8.6)	33 (12.0)	15 (16.3)	-	
	Situational	102 (11.5)	48 (17.4)	6 (6.5)		
**RADIOLOGY REPORT, Importance given for each section (/5)**						**(Eta^2)**
	Title	2.10±1.93	2.74±1.91	3.07±1.83	**<0.001**	**0.030**
	Indication	2.48±1.71	2.77±1.70	2.97±1.86	<0.001	0.007
	Technique	0.97±1.25	1.66±1.43	1.68±1.50	**<0.001**	**0.063**
	Results	3.73±1.23	4.12±1.11	4.08±1.29	**<0.001**	**0.026**
	Conclusion	4.47±1.17	4.62±0.92	4.51±1.18	<0.001	<0.001
	Name	2.47±1.67	3.54±1.39	3.67±1.35	**<0.001**	**0.090**
**KEY IMAGES**						
	Notation (/5)	3.63±1.16	3.84±1.22	3.96±1.19	**<0.001**	**0.012**
Enhance report quality	Strongly agree<br>Agree<br>Undecided<br>Disagree<br>Strongly disagree	142 (16.0)<br>220 (24.9)<br>287 (32.4)<br>152 (17.2)<br>84 (9.5)	46 (16.7)<br>66 (23.9)<br>106 (38.4)<br>29 (10.5)<br>29 (10.5)	17 (18.5)<br>21 (22.8)<br>31 (33.7)<br>14 (15.2)<br>9 (9.8)	0.344	0.060
Avoid opening the imaging examination	Strongly agree<br>Agree<br>Undecided<br>Disagree<br>Strongly disagree	96 (10.8)<br>185 (20.9)<br>156 (17.6)<br>218 (24.6)<br>230 (26.0)	38 (13.8)<br>42 (15.2)<br>49 (17.8)<br>63 (22.8)<br>84 (30.4)	11 (12.0)<br>20 (21.7)<br>20 (21.7)<br>17 (18.5)<br>24 (26.1)	0.338	0.060
Would like their systematic inclusion in radiology report	Strongly agree<br>Agree<br>Undecided<br>Disagree<br>Strongly disagree	280 (31.6)<br>290 (32.8)<br>218 (24.6)<br>54 (6.1)<br>43 (4.9)	93 (33.7)<br>71 (25.7)<br>63 (22.8)<br>26 (9.4)<br>23 (8.3)	32 (34.8)<br>27 (29.3)<br>19 (20.7)<br>9 (9.8)<br>5 (5.4)	0.099	0.073
Help understanding the radiology report	Strongly agree<br>Agree<br>Undecided<br>Disagree<br>Strongly disagree	318 (35.9)<br>377 (42.6)<br>126 (14.2)<br>34 (3.8)<br>30 (3.4)	123 (44.6)<br>99 (35.9)<br>29 (10.5)<br>15 (5.4)<br>10 (3.6)	38 (41.3)<br>33 (35.9)<br>14 (15.2)<br>5 (5.4)<br>2 (2.2)	0.169	0.068
Reinforce confidence toward the radiologist's conclusion	Strongly agree<br>Agree<br>Undecided<br>Disagree<br>Strongly disagree	259 (29.3)<br>286 (32.3)<br>238 (26.9)<br>64 (7.2)<br>38 (4.3)	79 (28.6)<br>86 (31.2)<br>80 (29.0)<br>19 (6.9)<br>12 (4.3)	24 (26.1)<br>24 (26.1)<br>30 (32.6)<br>8 (8.7)<br>6 (6.5)	0.878	0.039
**USE of THE RADIOLOGY REPORT**						
Notation of the radiologist's diagnostic hypothesis (/5)		4.03±0.70	3.92±0.75	3.89±0.84	0.082	0.002
Do you follow radiologist's recommendation for additional imaging?	Always<br>Often<br>Sometimes<br>Rarely<br>Never	188 (21.2)<br>588 (66.4)<br>101 (11.4)<br>8 (0.9)<br>0	61 (22.1)<br>171 (62.0)<br>35 (12.7)<br>6 (2.2)<br>3 (1.0)	24 (26.1)<br>51 (55.4)<br>35 (12.7)<br>4 (4.3)<br>0	0.004	0.095
How do you consider the radiologist's medical care recommendation?	It is necessary<br>It has an additional value<br>Not his/her role<br>Do not follow	86 (9.7)<br>579 (65.4)<br>170 (19.2)<br>50 (5.6)	18 (6.5)<br>170 (61.6)<br>76 (27.5)<br>23 (8.4)	5 (5.4)<br>51 (55.4)<br>31 (33.7)<br>5 (5.4)	0.002	0.112
Use of the RR within the medical record	Copy/Paste<br>Reformulate<br>Do not consider	551 (62.3)<br>305 (34.4)<br>29 (3.3)	120 (43.5)<br>134 (48.5)<br>22 (8.0)	27 (29.3)<br>56 (60.9)<br>9 (9.8)	**<0.001**	**0.158**
Have you ever made a mistake by not reading the RR?	Yes<br>No<br>Do not know	67 (7.6)<br>477 (53.9)<br>341 (38.5)	24 (8.7)<br>143 (51.8)<br>109 (39.5)	4 (4.4)<br>52 (56.5)<br>36 (39.1)	0.717	0.029
About the preliminary report, do you read it?	Yes, consider it definitive<br>Yes, but wait for senior validation<br>Yes, but call if disagree<br>No, wait for senior validation<br>Not concerned	187 (21.1)<br>413 (46.7)<br>273 (30.8)<br>12 (1.4)<br>0	28 (10.1)<br>157 (56.9)<br>73 (26.4)<br>9 (3.3)<br>9 (3.3)	8 (8.7)<br>56 (60.9)<br>17 (18.5)<br>6 (6.5)<br>5 (5.4)	**<0.001**	**0.211**
In a vital emergency, what is your attitude toward the RR	You watch directly images<br>Do not wait, ask specialist to watch<br>Wait final RR<br>Not concerned	429 (48.5)<br>171 (19.3)<br>44 (4.9)<br>241 (27.2)	154 (55.8)<br>41 (14.9)<br>7 (2.5)<br>74 (26.8)	43 (46.7)<br>20 (21.7)<br>4 (4.3)<br>25 (27.2)	0.228	0.057
Do you read the RR post-emergency?	Yes<br>No<br>Not concerned	678 (76.6)<br>59 (6.7)<br>148 (16.7)	196 (71.0)<br>30 (10.9)<br>50 (18.1)	67 (72.8)<br>7 (7.6)<br>18 (19.6)	0.175	0.050

Supplemental Table 2. Responses depending on age category
Bolded *P* values and effect size results indicate an effect size>0.100 (Cramer V, for proportions), or >0.0100 ($Eta^{2,}$ for numerical data)

		ACADEMIC STATUS			
		**Academic n=745 (59.5)**	**Non-Academic n=508 (40.5)**	***P* value**	**Effect Size**
**RADIOLOGY REPORT Reading pattern**					
	Integrality	200 (26.8)	157 (30.9)	**<0.001**	**0.149**
	Results depending on conclusion	212 (28.5)	186 (36.6)	-	
	Conclusion	130 (17.4)	88 (17.3)	-	
	Images only	89 (11.9)	35 (6.9)	-	
	Situational	114 (15.3)	42 (8.3)		
**RADIOLOGY REPORT, Importance given for each section (/5)**					
	Title	2.08±1.93	2.65±1.93	**<0.001**	**0.145**
	Indication	2.35±1.71	2.90±1.70	**<0.001**	**0.157**
	Technique	1.17±1.34	1.17±1.37	<0.001	0.006
	Results	3.88±1.18	3.80±1.26	<0.001	0.020
	Conclusion	4.50±1.11	4.51±1.14	<0.001	0.028
	Name	2.86±1.60	2.69±1.75	<0.001	0.045
**KEY IMAGES**					
	Notation (/5)	3.84±1.12	3.49±1.23	**<0.001**	**0.148**
Help understanding the radiology report	Strongly agree<br>Agree<br>Undecided<br>Disagree<br>Strongly disagree	317 (42.5)<br>310 (41.6)<br>82 (11.1)<br>22 (2.9)<br>14 (1.8)	162 (31.9)<br>199 (39.2)<br>87 (17.1)<br>32 (6.3)<br>28 (5.5)	**<0.001**	**0.173**
Enhance report quality	Strongly agree<br>Agree<br>Undecided<br>Disagree<br>Strongly disagree	125 (16.7)<br>196 (26.3)<br>258 (34.6)<br>107 (14.3)<br>59 (7.9)	80 (15.0)<br>111 (21.9)<br>166 (32.7)<br>88 (17.3)<br>63 (12.4)	<0.001	0.093
Avoid opening the imaging examination	Strongly agree<br>Agree<br>Undecided<br>Disagree<br>Strongly disagree	68 (9.1)<br>119 (15.9)<br>136 (18.2)<br>198 (26.5)<br>224 (30.1)	77 (15.2)<br>128 (25.2)<br>89 (17.5)<br>100 (19.7)<br>114 (22.4)	**<0.001**	**0.168**
Would like their systematic inclusion in radiology report	Strongly agree<br>Agree<br>Undecided<br>Disagree<br>Strongly disagree	243 (32.6)<br>229 (30.7)<br>184 (24.7)<br>56 (7.5)<br>33 (4.4)	162 (31.8)<br>159 (31.3)<br>116 (22.8)<br>33 (6.5)<br>38 (7.4)	<0.001	0.069
Reinforce confidence toward the radiologist's conclusion	Strongly agree.<br>Agree.<br>Undecided<br>Disagree<br>Strongly disagree	237 (31.8)<br>237 (31.8)<br>207 (27.7)<br>45 (6.1)<br>19 (2.5)	125 (24.6)<br>159 (31.3)<br>141 (27.7)<br>46 (9.1)<br>37 (7.2)	**<0.001**	**0.139**
**USE of THE RADIOLOGY REPORT**					
Notation of the radiologist's diagnostic hypothesis (/5)		3.93±0.70	4.08±0.75	**<0.001**	**0.206**
Do you follow radiologist's recommendation for additional imaging?	Always<br>Often<br>Sometimes<br>Rarely<br>Never	135 (18.1)<br>514 (68.9)<br>87 (11.6)<br>9 (1.7)<br>0	138 (27.1)<br>296 (58.2)<br>62 (12.2)<br>9 (1.7)<br>3 (0.5)	**<0.001**	**0.132**
How do you consider the radiologist's medical care recommendation?	It is necessary<br>It has an additional value<br>Not his/her role<br>Do not follow	15 (2.1)<br>473 (63.4)<br>132 (17.7)<br>34 (4.5)	26 (5.1)<br>327 (64.3)<br>73 (14.3)<br>14 (2.7)	**<0.001**	**0.152**
Use of the RR within the medical record	Copy/Paste<br>Reformulate<br>Do not consider	414 (55.5)<br>293 (39.3)<br>38 (5.1)	284 (55.9)<br>202 (39.7)<br>22 (4.3)	<0.001	0.018
Have you ever made a mistake by not reading the RR?	Yes<br>No<br>Do not know	60 (8.1)<br>403 (54.1)<br>282 (37.9)	35 (6.9)<br>269 (53.0)<br>204 (40.2)	0.598	0.029
About the preliminary report, do you read it?	Yes, consider it definitive<br>Yes, but wait for senior validation<br>Yes, but call if disagree<br>No, wait for senior validation<br>Not concerned	79 (10.6)<br>276 (37.1)<br>165 (22.1)<br>9 (1.2)<br>1 (0.1)	82 (16.1)<br>238 (46.8)<br>80 (15.7)<br>18 (3.5)<br>13 (2.5)	**<0.001**	**0.241**
In a vital emergency, what is your attitude toward the RR	You watch directly images<br>Do not wait, ask specialist to watch<br>Wait final RR<br>Not concerned	426 (57.1)<br>145 (19.4)<br>26 (3.4)<br>148 (19.8)	200 (39.4)<br>87 (17.1)<br>29 (5.7)<br>192 (37.8)	**<0.001**	**0.217**
Do you read the RR post-emergency?	Yes<br>No<br>Not concerned	586 (78.6)<br>65 (8.7)<br>94 (12.6)	31 (6.1)<br>355 (69.9)<br>122 (24.0)	**<0.001**	**0.151**

Supplemental Table 3. Responses depending on academic status (Academic *versus* Non-Academic)

Bolded *P* values and effect size results indicate an effect size>0.100

Supplemental Tables 4. Detailed results for medical specialities, summary for specialities above 30 respondents

Question : About CT and MRI radiology reports, **do you read**?

Reading strategy	Medical group n=1064 (85%)	General med. n=306 (28.8%)	Emergency med. n=99 (9.3%)	Anesthesiol. n=85 (8%)	Pediatrics n=70 (6.6%)	Oncology n=69 (6.5%)	Neurology n=42 (3.9%)	Cardiology n=33 (3.1%)	Rheumatology n=30 (2.8%)
Integrality	321 (30.2%)	93 (30.4%)	20 (20.2%)	28 (32.9%)	25 (35.7%)	17 (24.6%)	11 (26.2%)	11 (33.3%)	6 (20%)
Results*	363 (34.1%)	143 (46.7%)	32 (32.3%)	19 (22.4%)	25 (35.7%)	24 (34.8%)	6 (14.3%)	11 (33.3%)	7 (23.3%)
Conclusion	189 (17.8%)	57 (18.6%)	24 (24.2%)	19 (22.4%)	15 (21.4%)	13 (18.8%)	6 (14.3%)	7 (21.2%)	2 (6.7%)
Images only	0 (0%)	0 (0%)	0 (0%)	0 (0%)	0 (0%)	0 (0%)	0 (0%)	0 (0%)	0 (0%)
Situational	131 (12.3%)	9 (2.9%)	16 (16.2%)	15 (17.6%)	4 (5.7%)	8 (11.6%)	14 (33.3%)	2 (6.1%)	11 (36.7%)

*The main body of the report ("Results", depending on the conclusion)

Question: How important do you consider the **different sections of the report**?
0-5 score: from 0: if you generally do not read to 5: if you always read

Results score	Medical group n=1064 (85%)	General med. n=306 (28.8%)	Emergency med. n=99 (9.3%)	Anesthesiol. n=85 (8%)	Pediatrics n=70 (6.6%)	Oncology n=69 (6.5%)	Neurology n=42 (3.9%)	Cardiology n=33 (3.1%)	Rheumatology n=30 (2.8%)
0	3 (0.3%)	1 (0.3%)	0 (0%)	0 (0%)	0 (0%)	0 (0%)	0 (0%)	1 (3%)	0 (0%)
1	54 (5.1%)	25 (8.2%)	5 (5.1%)	1 (1.2%)	3 (4.3%)	0 (0%)	0 (0%)	2 (6.1%)	1 (3.3%)
2	111 (10.4%)	27 (8.8%)	15 (15.2%)	11 (12.9%)	8 (11.4%)	3 (4.3%)	3 (7.1%)	5 (15.2%)	7 (23.3%)
3	146 (13.7%)	51 (16.7%)	12 (12.1%)	6 (7.1%)	7 (10%)	7 (10.1%)	7 (16.7%)	2 (6.1%)	5 (16.7%)
4	346 (32.5%)	108 (35.3%)	35 (35.4%)	25 (29.4%)	17 (24.3%)	28 (40.6%)	18 (42.9%)	13 (39.4%)	11 (36.7%)
5	404 (38%)	94 (30.7%)	32 (32.3%)	42 (49.4%)	35 (50%)	31 (44.9%)	14 (33.3%)	10 (30.3%)	6 (20%)

Conclusion score	Medical group n=1064 (85%)	General med. n=306 (28.8%)	Emergency med. n=99 (9.3%)	Anesthesiol. n=85 (8%)	Pediatrics n=70 (6.6%)	Oncology n=69 (6.5%)	Neurology n=42 (3.9%)	Cardiology n=33 (3.1%)	Rheumatology n=30 (2.8%)
0	0 (0%)	0 (0%)	0 (0%)	0 (0%)	0 (0%)	0 (0%)	0 (0%)	0 (0%)	0 (0%)
1	26 (2.4%)	6 (2%)	3 (3%)	2 (2.4%)	2 (2.9%)	0 (0%)	0 (0%)	0 (0%)	2 (6.7%)
2	117 (11%)	37 (12.1%)	16 (16.2%)	9 (10.6%)	9 (12.9%)	1 (1.4%)	2 (4.8%)	5 (15.2%)	5 (16.7%)
3	5 (0.5%)	0 (0%)	0 (0%)	0 (0%)	0 (0%)	0 (0%)	0 (0%)	0 (0%)	0 (0%)
4	29 (2.7%)	3 (1%)	1 (1%)	1 (1.2%)	0 (0%)	3 (4.3%)	2 (4.8%)	1 (3%)	4 (13.3%)
5	887 (83.4%)	260 (85%)	79 (79.8%)	73 (85.9%)	59 (84.3%)	65 (94.2%)	38 (90.5%)	27 (81.8%)	19 (63.3%)

Name score	Medical group n=1064 (85%)	General med. n=306 (28.8%)	Emergency med. n=99 (9.3%)	Anesthesiol. n=85 (8%)	Pediatrics n=70 (6.6%)	Oncology n=69 (6.5%)	Neurology n=42 (3.9%)	Cardiology n=33 (3.1%)	Rheumatology n=30 (2.8%)
0	129 (12.1%)	62 (20.3%)	13 (13.1%)	5 (5.9%)	6 (8.6%)	2 (2.9%)	1 (2.4%)	2 (6.1%)	3 (10%)
1	183 (17.2%)	79 (25.8%)	13 (13.1%)	10 (11.8%)	10 (14.3%)	8 (11.6%)	4 (9.5%)	4 (12.1%)	3 (10%)
2	151 (14.2%)	42 (13.7%)	19 (19.2%)	11 (12.9%)	6 (8.6%)	13 (18.8%)	2 (4.8%)	6 (18.2%)	4 (13.3%)
3	212 (19.9%)	51 (16.7%)	20 (20.2%)	23 (27.1%)	17 (24.3%)	19 (27.5%)	12 (28.6%)	7 (21.2%)	8 (26.7%)
4	199 (18.7%)	32 (10.5%)	16 (16.2%)	21 (24.7%)	14 (20%)	18 (26.1%)	10 (23.8%)	4 (12.1%)	5 (16.7%)
5	190 (17.9%)	40 (13.1%)	18 (18.2%)	15 (17.6%)	17 (24.3%)	9 (13%)	13 (31%)	10 (30.3%)	7 (23.3%)

Question: Once the radiology report is obtained, **how do you use it in your medical observation**?

Observation	Medical group n=1064 (85%)	General med. n=306 (28.8%)	Emergency med. n=99 (9.3%)	Anesthesiol. n=85 (8%)	Pediatrics n=70 (6.6%)	Oncology n=69 (6.5%)	Neurology n=42 (3.9%)	Cardiology n=33 (3.1%)	Rheumatology n=30 (2.8%)
Copy/paste	633 (59.5%)	190 (62.1%)	88 (88.9%)	53 (62.4%)	41 (58.6%)	24 (34.8%)	20 (47.6%)	18 (54.5%)	14 (46.7%)
Reformulate	409 (38.4%)	115 (37.6%)	10 (10.1%)	31 (36.5%)	29 (41.4%)	40 (58%)	19 (45.2%)	14 (42.4%)	12 (40%)
Do not take into account	22 (2.1%)	1 (0.3%)	1 (1%)	1 (1.2%)	0 (0%)	5 (7.2%)	3 (7.1%)	1 (3%)	4 (13.3%)

Question: When a resident publishes a **preliminary report**, do you read it?

Preliminary report	Medical group n=1064 (85%)	General med. n=306 (28.8%)	Emergency med. n=99 (9.3%)	Anesthesiol. n=85 (8%)	Pediatrics n=70 (6.6%)	Oncology n=69 (6.5%)	Neurology n=42 (3.9%)	Cardiology n=33 (3.1%)	Rheumatology n=30 (2.8%)
Yes, but wait for senior validation	449 (45.6%)	143 (50.7%)	20 (23.8%)	30 (37%)	36 (52.9%)	31 (50%)	19 (45.2%)	21 (70%)	12 (41.4%)
Yes, but call if disagree	184 (18.7%)	41 (14.5%)	24 (28.6%)	21 (25.9%)	7 (10.3%)	16 (25.8%)	11 (26.2%)	5 (16.7%)	4 (13.8%)
Yes, definitive	146 (14.8%)	68 (24.1%)	22 (26.2%)	5 (6.2%)	1 (1.5%)	6 (9.7%)	3 (7.1%)	1 (3.3%)	3 (10.3%)
if disagree; Yes, but wait for sen	206 (20.9%)	30 (10.6%)	18 (21.4%)	25 (30.9%)	24 (35.3%)	9 (14.5%)	9 (21.4%)	3 (10%)	10 (34.5%)

Questions: How important do you **consider key images**? 0 to 5 score: From 0: not important to 5: very important
What is your level of confidence in the **radiologist's diagnostic hypotheses**? (1 to 5 score)

	Medical group	General medicine	Emergency medicine	Anesthesiology	Pediatrics	Oncology	Neurology	Cardiology	Rheumatology
**Key Images' importance**	3.71 ± 1.17	3.31 ± 1.19	3.79 ± 1.07	3.79 ± 1.1	4.2 ± 0.81	3.97 ± 1.2	4.1 ± 0.88	3.12 ± 1.41	3.6 ± 1.25
**Radiologist's hypothesis**	4.06 ± 0.68	4.22 ± 0.6	4.16 ± 0.68	3.94 ± 0.79	4.23 ± 0.57	3.97 ± 0.75	3.93 ± 0.71	3.76 ± 0.75	3.67 ± 0.84

Question: **Have you ever made a medical mistake** due to not thoroughly reading the radiology report?

Mistake	Medical group n=1064 (85%)	General med. n=306 (28.8%)	Emergency med. n=99 (9.3%)	Anesthesiol. n=85 (8%)	Pediatrics n=70 (6.6%)	Oncology n=69 (6.5%)	Neurology n=42 (3.9%)	Cardiology n=33 (3.1%)	Rheumatology n=30 (2.8%)
Do not know	415 (39%)	134 (43.8%)	31 (31.3%)	30 (35.3%)	20 (28.6%)	23 (33.3%)	15 (35.7%)	9 (27.3%)	11 (36.7%)
No	568 (53.4%)	158 (51.6%)	59 (59.6%)	47 (55.3%)	49 (70%)	36 (52.2%)	25 (59.5%)	22 (66.7%)	17 (56.7%)
Yes	81 (7.6%)	14 (4.6%)	9 (9.1%)	8 (9.4%)	1 (1.4%)	10 (14.5%)	2 (4.8%)	2 (6.1%)	2 (6.7%)

Question : Do you usually follow the **radiologist's recommendations regarding additional imaging** proposals?

Complementary imaging	Medical group n=1064 (85%)	General med. n=306 (28.8%)	Emergency med. n=99 (9.3%)	Anesthesiol. n=85 (8%)	Pediatrics n=70 (6.6%)	Oncology n=69 (6.5%)	Neurology n=42 (3.9%)	Cardiology n=33 (3.1%)	Rheumatology n=30 (2.8%)
Always	256 (24.1%)	88 (28.8%)	23 (23.2%)	18 (21.2%)	18 (25.7%)	13 (18.8%)	9 (21.4%)	5 (15.2%)	6 (20%)
Often	686 (64.5%)	191 (62.4%)	68 (68.7%)	53 (62.4%)	49 (70%)	40 (58%)	30 (71.4%)	23 (69.7%)	19 (63.3%)
Sometimes	113 (10.6%)	26 (8.5%)	7 (7.1%)	14 (16.5%)	3 (4.3%)	13 (18.8%)	3 (7.1%)	5 (15.2%)	3 (10%)
Rarely	9 (0.8%)	1 (0.3%)	1 (1%)	0 (0%)	0 (0%)	3 (4.3%)	0 (0%)	0 (0%)	2 (6.7%)

Statistical analysis for differences between main medical subspecialities demonstrated a statistically significant difference for each parameter, with a *P* value <0.001, except for Conclusion-rating with P value <0.05.
Fischer test was performed for categorical variables, and Kruskal-Wallis test for ordinal score variables.
